# Synthesis, docking, MD simulation, ADMET, drug likeness, and DFT studies of novel furo[2,3-*b*]indol-3a-ol as promising Cyclin-dependent kinase 2 inhibitors

**DOI:** 10.1038/s41598-024-53514-1

**Published:** 2024-02-07

**Authors:** Davood Gheidari, Morteza Mehrdad, Mohammad Bayat

**Affiliations:** 1 Department of Chemistry, Faculty of Science, University of Guilan, Rasht, Iran; 2 Department of Chemistry, Faculty of Science, Imam Khomeini International University, Qazvin, Iran

**Keywords:** Chemistry, Cheminformatics

## Abstract

A new series of furo[2,3-*b*]indol-3*a*-ol derivatives was synthesized to investigate their potential as inhibitors of the Cyclin-dependent kinase 2 (CDK2) enzyme. CDK2 is a serine/threonine protein kinase belonging to a family of kinases involved in the control of the cell cycle. Based on results from clinical studies, it has been shown that overexpression of CDK2 may play a role in the development of cancer. In order to discover highly effective derivatives, a process of in *silico* screening was carried out. The obtained results revealed that compound **3f.** had excellent binding energies. In this study, in *silico* screening was used to investigate protein–ligand interactions and assess the stability of the most favorable conformation. The methods utilized included molecular docking, density functional theory (DFT) calculations using the B3LYP/6-31++G(d,p) basis set in the gas phase, molecular dynamic (MD) simulation, as well as the evaluation of drug-likeness scores. The pharmacokinetic and drug-likeness properties of the novel furo[2,3-*b*]indol-3a-ol derivatives suggest that these compounds have the potential to be considered viable candidates for future development as anticancer drugs.

## Introduction

**Figure 1:**
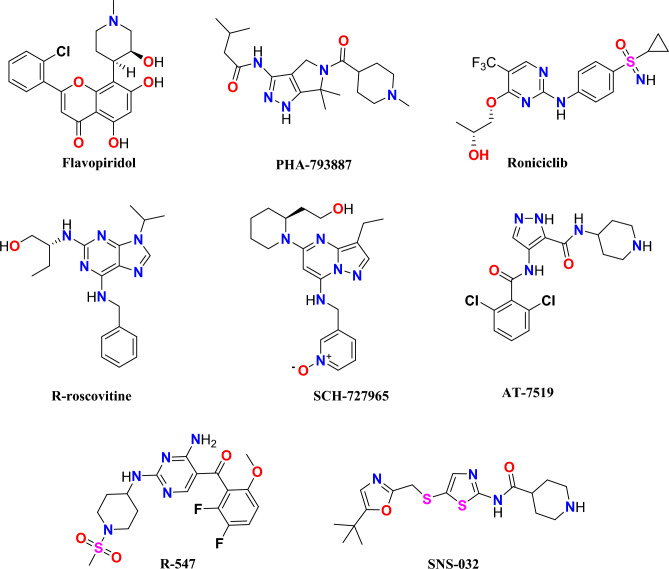
Structure of CDK2 inhibitors.

Cyclin-dependent kinase (CDK) is a serine/threonine protein kinase family with a total of 20 members, including CDK1-CDK20^1^. The CDK family associates with cyclin and plays a vital role in controlling the cell cycle^2^. CDKs and cyclins are frequently observed to be upregulated in neoplastic cells. Hence, inhibitors of CDKs have been identified as potential therapeutic agents for the treatment of cancer^3,4^. CDK2 holds significant importance as a member of the CDK family, as evidenced by studies conducted by Whittaker et al.^5^. CDK2 plays a pivotal role in the regulation of the cell cycle within actively dividing cells, exhibiting significant functionality during the latter part of the G1 phase and throughout the entire S phase^6^. According to clinical studies, the elevation in CDK2 activity has been identified as a potential factor contributing to the onset of malignancies. Furthermore, a substantial body of evidence suggests that the activity of CDK2 has an impact on both cell differentiation and adaptive immunological responses^7–9^. Hence, the proliferation of cancer cells can be hindered through the direct binding of CDK2 and cyclin by CDK2 inhibitors^10^. The varied functions of CDK2 within cellular proliferation and survival pathways render it a highly compelling target for mechanism-driven and low-toxicity therapeutic approaches in the field of cancer treatment^11^. Over the past two decades, several CDK2 inhibitors have undergone assessment in clinical trials. As shown in Fig. 1, the initial cohort included Flavopiridol, PHA-793887, SNS-032, and R-roscovitine^12^, while the subsequent group included SCH-727965, AT-7519, R-547, and roniciclib^13^. In light of the above information and within the context of our current scientific investigation aimed at the advancement of innovative and biologically active *N*-heterocycles, our research efforts have been directed towards the synthesis of a novel series of furo[2,3-*b*]indol-3a-ol derivatives. In this study, our objective was to utilize molecular docking, MD simulation, ADMET analysis, and DFT analysis to evaluate the drug-likeness of these compounds as potential building blocks for CDK2 inhibitors.

## Results and discussion

### Chemistry

We have devised a straightforward and effective method for synthesizing furo[2,3-*b*]indol-3a-ol derivatives **3a–f** via a one-pot reaction. To get the best reaction conditions, isatin **1a** (0.5 mmol) and *N*-methyl-1-(methylthio)-2-nitroethenamine **2** (0.5 mmol) were mixed together and refluxed in various solvents without any catalysts. Our results showed that ethanol is an effective solvent, resulting in the production of **3a** with a yield of 20% (entry 3, Table 1). Subsequently, the basic catalysts, namely Et_3_N and Cs_2_CO_3_, as well as the acid catalysts, specifically AcOH, *p-*TSA, and NH_2_SO_3_H, were employed in the same reaction, which underwent reflux in ethanol for a duration of 24 h (entries 4−8, Table 1). Various ratios of ethanol and water solvents were tested, revealing that a ratio of EtOH/H_2_O=1:3 led to the production of the target compound with a 90% yield. We investigated the reaction of *N*-methyl-1-(methylthio)-2-nitroethenamine **2** with various isatins **3a–f** to synthesize the desired compounds **3a–f** under optimized conditions. The reaction with other isatins, such as *N*-alkyl isatin derivatives and 4-chloroisatin, was conducted under the same conditions but did not yield the desired products.

**Table 1:** Optimize reaction conditions for the synthesis of 3a^a^


Entry	Solvent	Catalyst	Time (h)	Yield (%)
1	H_2_O		24	nr
2	toluene		24	Trace
3	EtOH		24	15
4	EtOH	NEt_3_	24	nr
5	EtOH	Cs_2_CO_3_	24	nr
6	EtOH	*p-*TSA	24	20
7	EtOH	AcOH	24	22
8	EtOH	NH_2_SO_3_H	24	33
9	H_2_O/EtOH(1:1)	NH_2_SO_3_H	24	51
10	H_2_O/EtOH(2:1)	NH_2_SO_3_H	24	60
11	**H**_**2**_**O/EtOH(3:1)**	**NH**_**2**_**SO**_**3**_**H**	**24**	**90**

^a^Reagents and conditions: **1a** (0.5 mmol), **2** (0.5 mmol), catalyst (0.05 mmol), solvent (4.0 mL). nr=no reaction.

**Figure 2:**
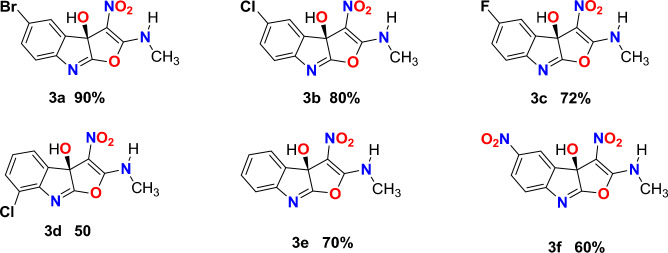
The Molecular structures and percent yields of the final compounds **3a–f**.

The comprehensive structures of the synthesized derivatives are illustrated in Fig. 2. The structures of compounds **3a–f** were determined based on the spectroscopic data obtained from Mass, IR, ^1^H NMR, and ^13^C NMR. For instance, the ^1^H NMR spectra of compound **3a** exhibited the presence of hydroxyl (OH) groups (δ 13.05 ppm) and an amine (NH) group (δ 9.07 ppm), which showed exchangeability with D_2_O. The NH proton exhibited a low-field shift due to intramolecular hydrogen bonding. The N–CH_3_ signal was observed at 2.83 ppm. The ^1^H-decoupled ^13^C NMR spectrum of **3a** exhibited 11 discernible resonances, confirming the proposed structure. The N–CH_3_ and C–OH displayed distinctive signals at δ 26.5 and 115.8 ppm, respectively. Three chemical shifts were observed at δ 116.2, 153.9, and 161.3 ppm, corresponding to C–NO_2_, C=N, and C-N groups, respectively. The mass spectrum of compound **3a** showed a molecular-ion peak at m/z 324, consistent with the suggested structure. The IR spectrum of this compound revealed the presence of hydroxyl (OH) and amino (NH) groups, observed broadly at 3434 cm^−1^ and 3272 cm^−1^, respectively. Additionally, stretching vibrations of the CH groups were detected at 2925 cm^−1^ 1 and 2859 cm^−1^. Other notable bands were observed at wavenumbers of 1693 cm^−1^, 1534 cm^−1^, 1376 cm^−1^, and 1260 cm^−1^, which were associated with groups including C=N, C–N, NO_2_, C–O, and C–N, respectively.

**Scheme 1.**
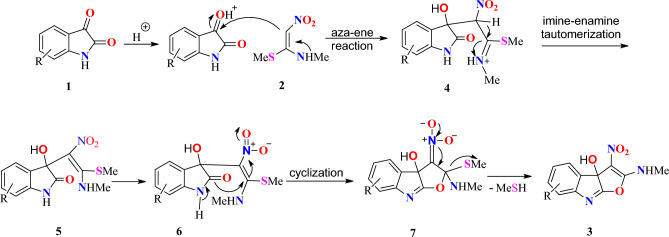
Suggested mechanism for the generation of product **3**.

A suggested mechanism for the generation of furo[2,3-*b*]indol-3a-ol **3** is illustrated in Scheme 1. The carbonyl group of isatin **1** is protonated in the first step. The nucleophilic attack of *N*-methyl-1-(methylthio)-2-nitroethenamine **2** upon the protonated carbonyl group of 1 by an aza-ene reaction affords compound **4**, which is converted to **5** by imine-enamine tautomerization. Consequently, the intramolecular annulation of compound **6** resulted in the formation of intermediate **7**. Ultimately, intermediate **7** was converted into the target product **3** by eliminating methanethiol.

### Computational studies

#### Quantum chemistry via density functional theory calculation

The values corresponding to each of the selected compounds **3a–f** are presented in Table 2. Initially, in the gas phase, the B3LYP/6-31++G(d,p) basis set was utilized in the Gaussian 09W software package and the Gauss View visualization tool to optimize these parameters^14^.

**Table 2:** Geometric parameters of the compounds **3a–f**.

S. No.	Compound	Gas phase		
		Optimization energy (hartree)	Polarizability (α) (a.u.)	Dipole moment (Debye)
1	**3a**	− 3460.7	196.45	6.54
2	**3b**	− 1349.2	187.09	6.59
3	**3c**	− 988.8	171.82	6.45
4	**3d**	− 1349.2	186.24	4.50
5	**3e**	− 889.6	171.78	4.66
6	**3f.**	− 1094.1	193.76	10.19

**Figure 3:**
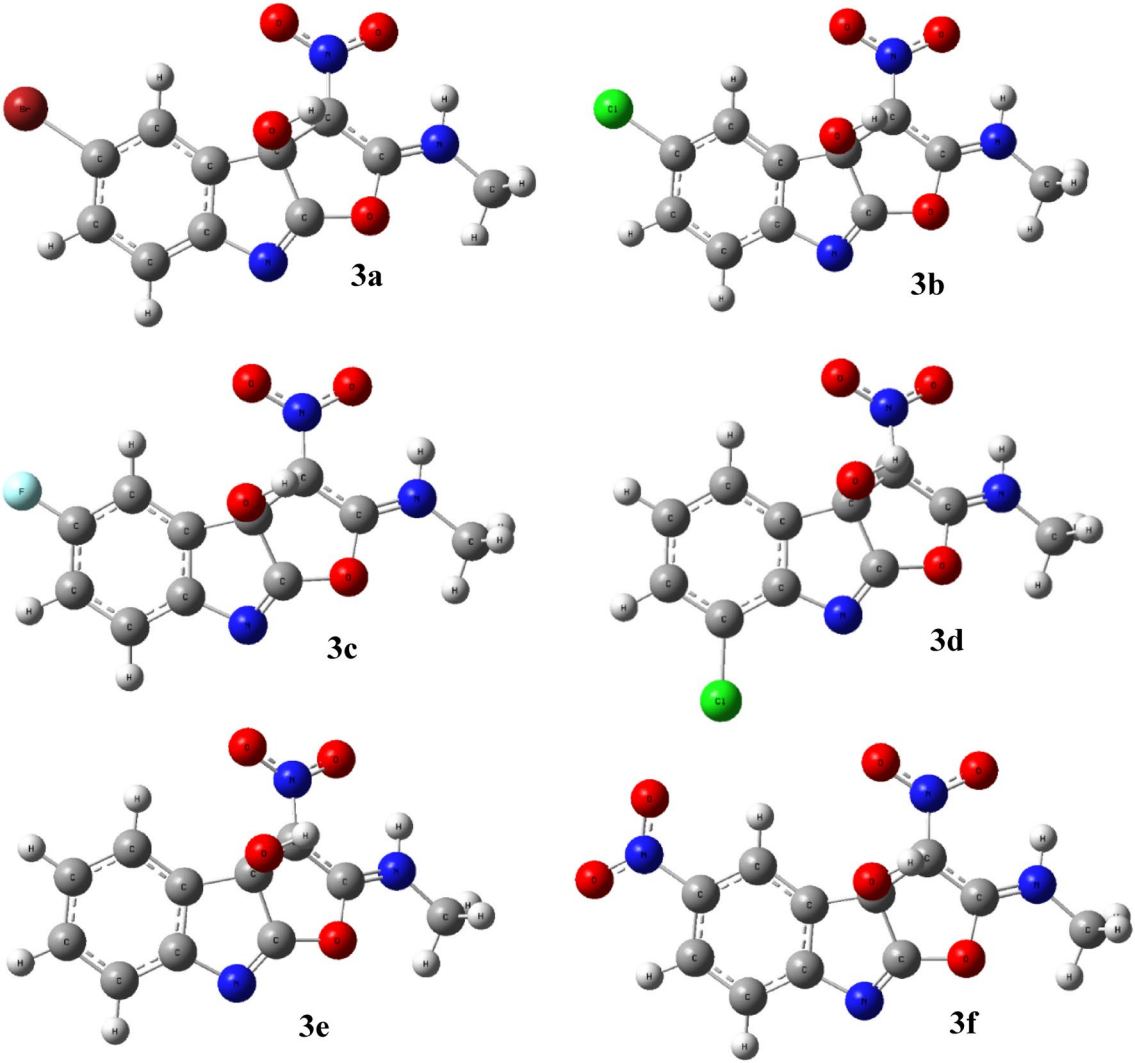
Optimized structures of the compounds **3a–f.**

There were no instances of imaginary frequencies found, and the geometries of the selected compounds were adjusted to lower energy gradients, indicating that all compounds were really local minima. Figure 3 shows the optimized structures of the selected compounds.

**Figure 4:**
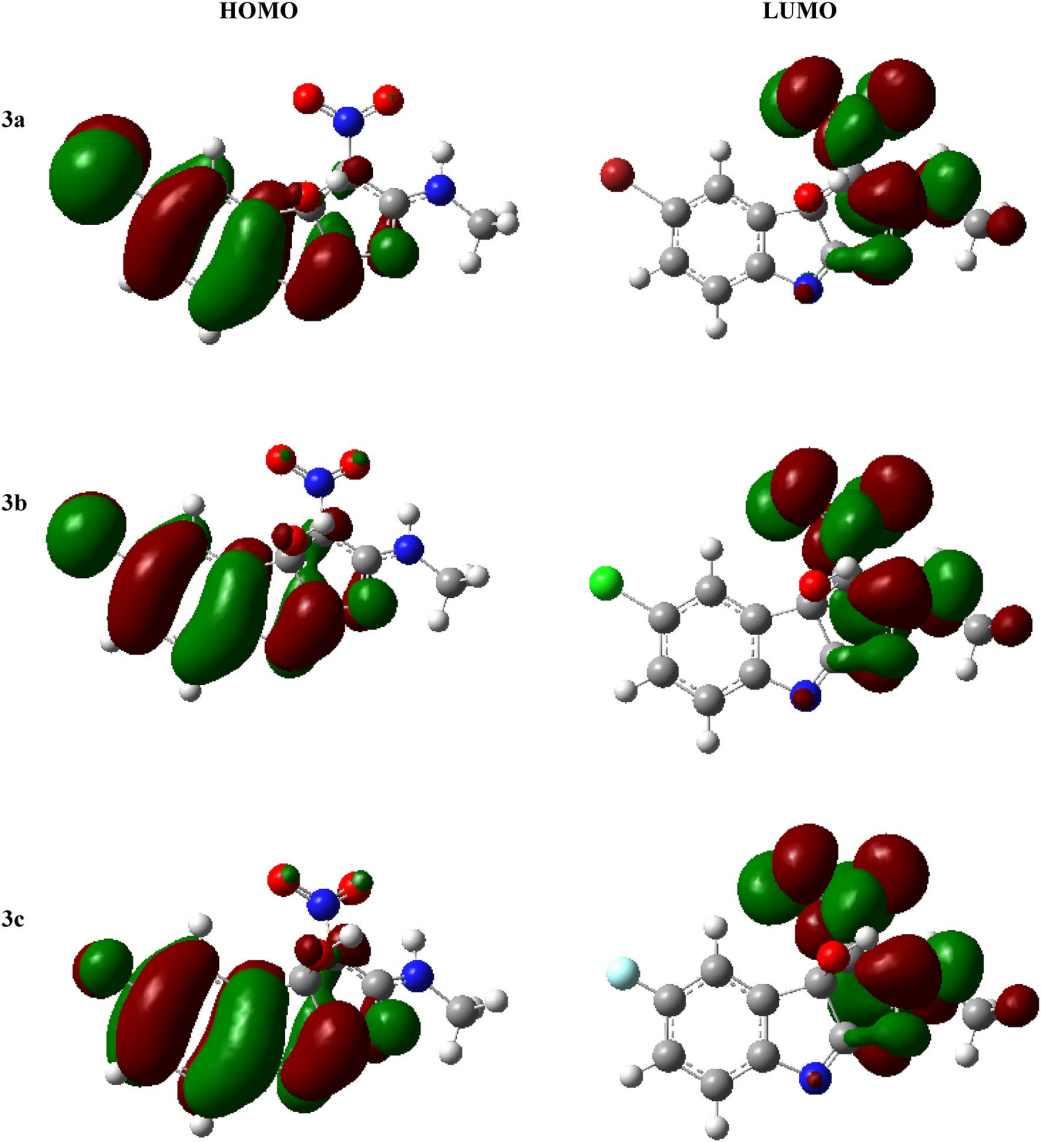

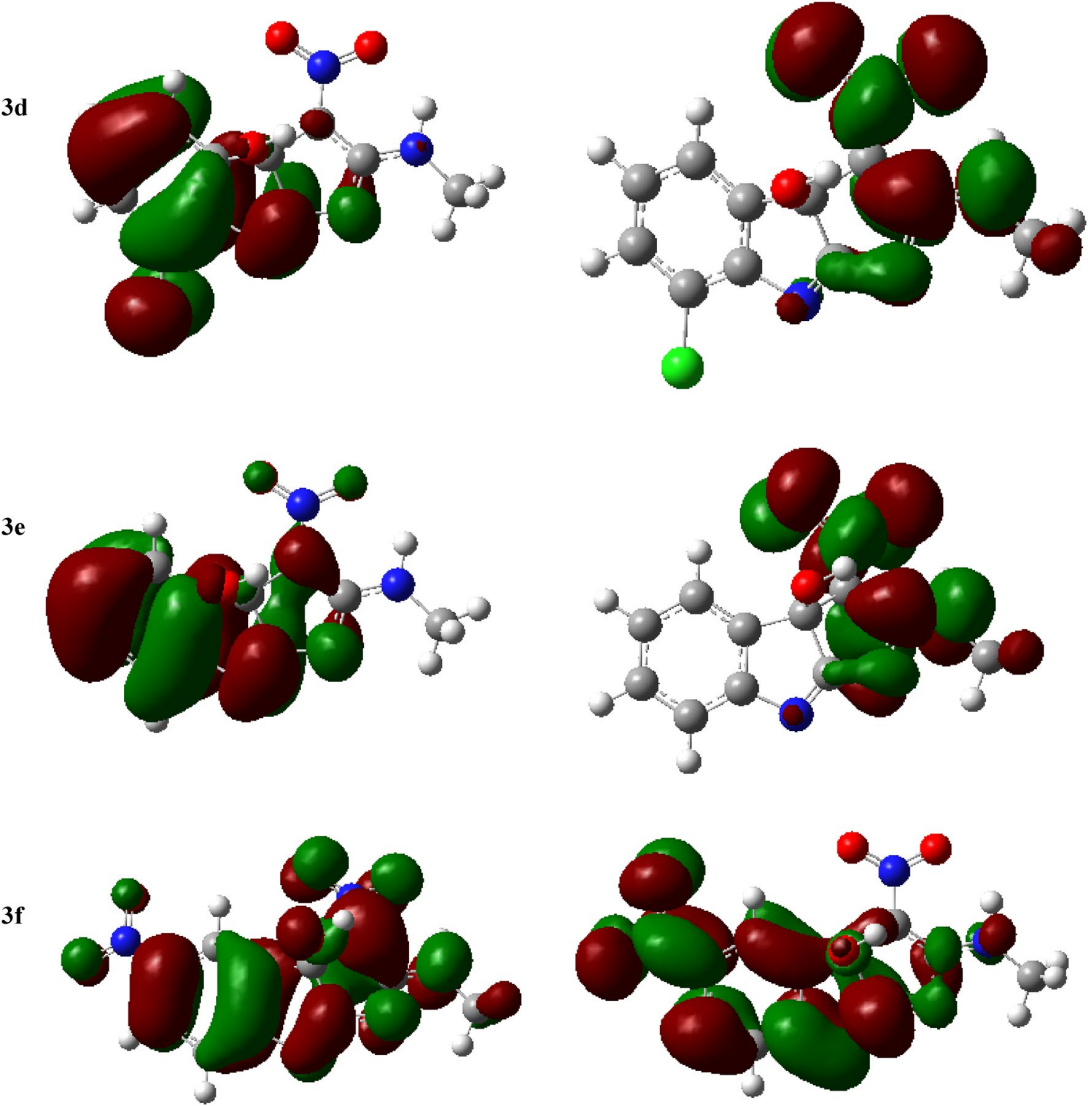
FMOs of the compounds **3a–f**.

The importance of Molecular Orbital Analysis (MO) in quantum chemistry is obvious since it serves as a pivotal tool for the comprehensive elucidation of chemical events. For the purpose of explaining chemical characteristics, the lowest unoccupied molecular orbital (LUMO) and the highest occupied molecular orbital (HOMO) are included. These qualities include reactivity, stability, and kinetics. The HOMO tends to release electrons, whereas the LUMO has a tendency to accept electrons. These orbitals can be utilized for assessing charge transfer phenomena. Indeed, the energy of the HOMO is related to ionization potential, while the energy of the LUMO is related to electron affinity. The frontier molecular orbitals (FMOs) of the synthesized compounds are illustrated in Fig. 4. The molecular orbital wave function attributes the positive and negative phases, respectively, to the color distributions of red and green.

The calculations conducted in the gas phase are presented in Table 3, encompassing various descriptors including HOMO–LUMO energies, band gap energies, chemical hardness (*η*), softness and potentials, electronegativity (*X*), and electrophilicity indexes. The parameters were computed using well-established approaches described in the literature, using the border orbital energies of HOMO and LUMO given in eV. The disparity in energy between the HOMO and the LUMO serves as a direct measure of chemical reactivity. A notable difference in energy between HOMO and LUMO signifies heightened stability and diminished chemical reactivity. Based on the results, compound **3a** has a low HOMO–LUMO energy gap of 4.114 eV, indicating a high degree of chemical reactivity. We can order this parameter in terms of ΔE_gap_ as follows: **3d > 3e > 3f. > e3c > 3b > 3a**. Furthermore, the maximum HOMO delocalization spans between 10 and 12 atoms in compounds **3a–3e**, whereas it is notably greater in compound **3f**. Conversely, the primary regions of LUMO delocalization in compounds **3a–3e** correspond to the furo, methylamino, and nitro groups. In compound **3f.**, almost all other groups contribute to delocalization, except for the nitro and methylamino groups, which play minimal roles. In addition, compound **3a** has the highest degree of softness among all compounds, as evidenced by its lowest recorded hardness value of 2.057 and its highest polarizability. Compound **3f.** exhibits a higher electronegativity value (5.231) compared to the other compounds, suggesting its enhanced ability to attract electrons and its improved performance as an electrophile (6.351). Compounds **3b** and **3c**, possessing energy gap values of 4.178 eV and 4.233 eV, respectively, exhibited notable reactivity subsequent to compound **3a**. Similarly, compounds **3f.**, **3b**, and **3d** exhibit significant polarizability subsequent to **3a**, with corresponding values of 193.76, 187.09, and 186.24. Table 3 presents the energy characteristics of compounds **3a-f**.

**Table 3:** Parameters of energy for compounds **3a–f**.

Compound	*E*_*HOMO*_ (eV)	*E*_*LUMO*_ (eV)	∆E _*gap*_ (eV)	Hardness (*η*)	Softness (*S*)	Electronegativity (*X*)	Electrophilicity ($\mathrm{c\psi }$)
3a	− 6.616	− 2.502	4.114	2.057	0.243	4.559	5.052
3b	− 6.678	− 2.500	4.178	2.089	0.239	4.589	5.044
3c	− 6.713	− 2.480	4.233	2.116	0.236	4.596	4.991
3d	− 6.846	− 2.498	4.348	2.174	0.229	4.672	5.020
3e	− 6.697	− 2.385	4.312	2.156	0.231	4.541	4.782
3f.	− 7.385	− 3.077	4.308	2.154	0.232	5.231	6.351

#### Molecular docking studies

CDK2 plays an important role in controlling the progression of the eukaryotic cell cycle. It is commonly known that monomeric CDK2 lacks inherent regulatory activity. Instead, its regulatory function is activated by positive regulators such as cyclins E and A or through phosphorylation on the catalytic section. It is noteworthy that the aforementioned activation processes elicit notable alterations in the three-dimensional configuration of the kinase, particularly in the activation section. The CDK2 protein consists of a solitary polypeptide chain with 306 amino acids. In fact, CDK2 has been identified as a significant contributor to the process of cell proliferation in prostate cancer^15^ and non-small cell carcinoma^16^. It has also been shown to have an important role in the malignant transformation of breast epithelial cells. Consequently, suppressing CDK2 activity has been proven to effectively impede the growth of cancer cells^17^. Considering the importance of CDK2, we conducted in *silico* investigations. The crystallographic structure of CDK2, identified by its Protein Data Bank (PDB) ID 6GUH^18^, was obtained at a resolution of 1.50. The amino acid residues that play a crucial role in the catalytic site have been identified and subjected to docking studies with potent furo[2,3-*b*]indol-3a-ol derivatives. The interactions with the amino acid residues present in the active pocket have been evaluated to determine the binding affinities and binding scores of the synthesized derivatives. The results revealed that a significant proportion of the compounds exhibited robust binding scores and remarkable binding affinities. Notably, compound **3f.** exhibited the best binding energy at − 6.89 kcal/mol. The best configuration of **3f.** was selected, followed by a comprehensive analysis of both bonding and non-bonding interactions. The docking results of the highly effective compounds, along with their corresponding interactions, are presented in Table 4.

**Table 4:** Docking scores and interaction for each compounds **3a–f**.

Compound	Docking scores	Types of interactions						
		H-bonding	C-bonding	Attractive Charge	*π*-alkyl	Alkyl	Van der walls	Unfavorable
**3a**	− 6.12	Gln139,Asp153	Gly21	Asp94	Phe88	Ala152,Ala39,Val26,Val72,Leu142	Lys41,Thr22,Glu20,Gly19,Asn140,Ile18,Glu89	Asp94
**3b**	− 6.34	Gln139,Asp153	Asn140	Asp94	Phe88	Ala152,Ala39,Val26,Val72,Leu142	Lys41,Thr22,Glu20,Gly19,Ile18,Glu89	Asp94
**3c**	− 6.78	Asp153	Gly21	Asp94	Phe88	Ala152,Ala39,Val26	Lys41,Thr22,Glu20,Gly19,Gln139,Asn140,Ile18,Leu142,Val72	Asp94
**3d**	− 5.95	Asp94,Ile18	Asp94,Ile18	Glu16,Lys97		Ile18	Gly19,Leu142,Leu91,Phe90,Gln93,His92,Gly19	Glu16, Ile18
**3e**	− 6.22	Gln139,Asp153	Gly21	Asp94	Phe88	Ala152,Ala39,Val26	Thr22,Lys41,Val72,Leu142,Ile18,Asn140,Gly19,Glu20	Asp94
**3f.**	− 6.89	Gln139,Asp94	Ile18,Gln139,Gly19,Asp153	Asp153		Leu142	Gln93,His92,Leu91,Ala39,Phe90,Val26,Ala152,Asn140,Gly21,Lys137,Thr22,Glu20	

**Figure 5:**
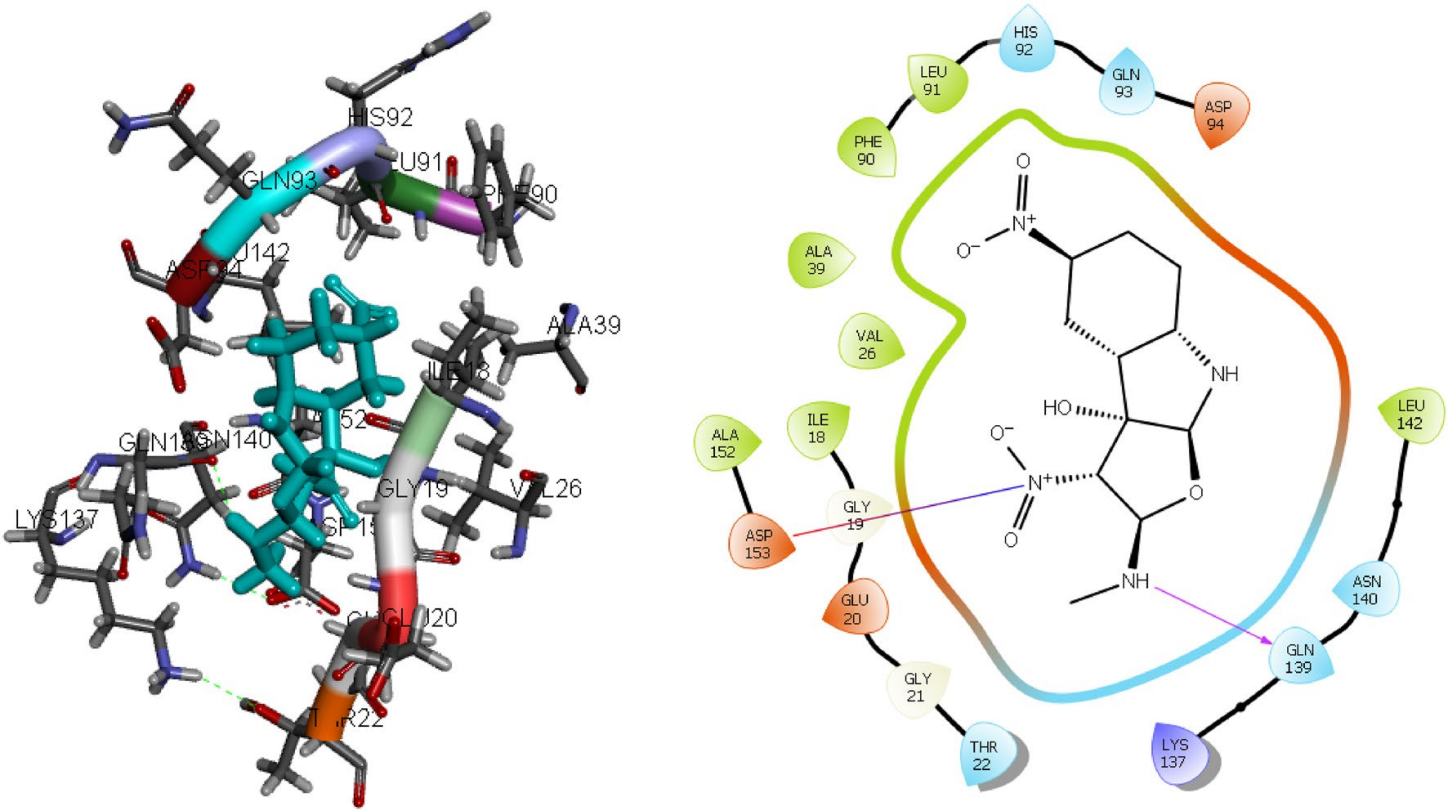
The 3D and 2D bindings mode of compound **3f.** into the active site of CDK2.

The detailed 3D and 2D binding interactions of compound **3f.** within the active pocket of CDK2 are shown in Fig. 5. The amino acid residues Gln139, Asp94, Ile18, Gln139, Gly19, Asp153, Leu142, Gln93, His92, Leu91, Ala39, Phe90, Val26, Ala152, Asn140, Gly21, Lys137, Thr22, and Glu20 were involved in both bonding and non-bonding interactions with compound **3f**. In summary, compound **3f.** exhibits a total of six hydrogen bond interactions, consisting of two conventional hydrogen bonds and four carbon hydrogen bonds. Also, it has an attractive charge with ASp153. Additionally, it has been demonstrated that compound **3f.** was responsible for creating one hydrophobic contact at the active site. Furthermore, the amino acid residues Gln93, His92, Leu91, Ala39, Phe90, Val26, Ala152, Asn140, Gly21, Lys137, Thr22, and Glu20 were found to be involved in the van der Waals interaction with compound **3f**.

**Figure 6:**
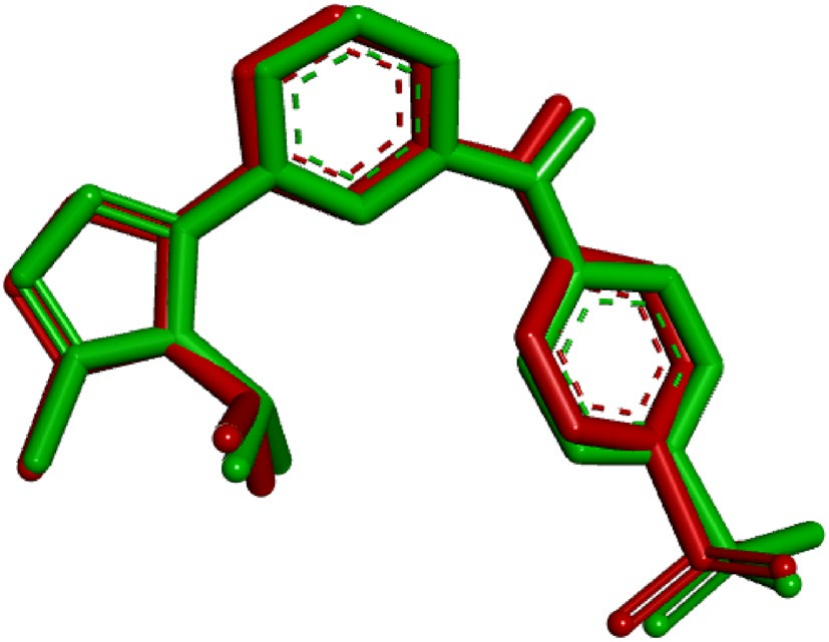
Superimposition of the docked ligand (red) and the original ligand (green).

To assess the validation of molecular docking, a re-docking procedure was conducted using the co-crystallized ligand. The resulting root-mean-square deviation (RMSD) value of 0.40 Å suggests that the docking experiment is reliable^19^ (Fig. 6).

#### Molecular dynamics simulation

**Figure 7:**
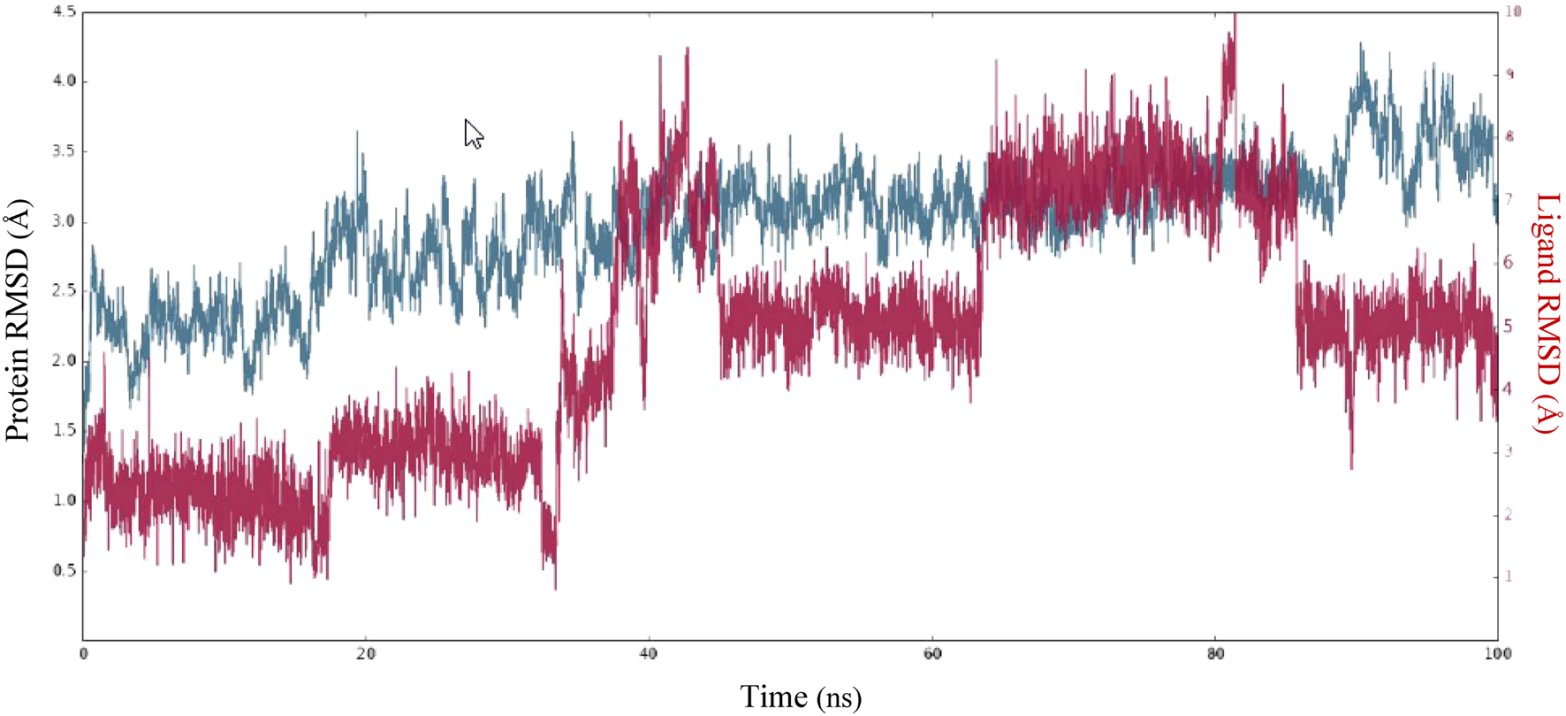
RMSD values of the protein and ligand during 100 ns MD simulation.

The molecular dynamics (MD) simulation of CDK2-ligand complexes was assessed over a time scale of 100 ns to elucidate the dynamic behavior and stability of the complexes. For this objective, a study was carried out to examine the structural alterations induced by the highly effective compound **3f**. The RMSD was examined during the 100 ns of MD simulation to assess the protein–ligand complex's stability. As illustrated in Fig. 7, the plot utilizes the left Y-axis to represent the RMSD of the protein and the right Y-axis to display the ligand RMSD profile that is aligned with the protein backbone. The frames obtained from the 100 ns trajectory were aligned with the reference frame backbone. The RMSD plot demonstrates the stability of the CDK2-ligand complex after 5 ns, relative to the reference frame formed at time point 0 ns. Nevertheless, a slight elevation in the RMSD of the protein-bound ligand was observed at 81 ns. This deviation might be attributed to a conformational change in the rotatable bonds of the ligand.

**Figure 8:**
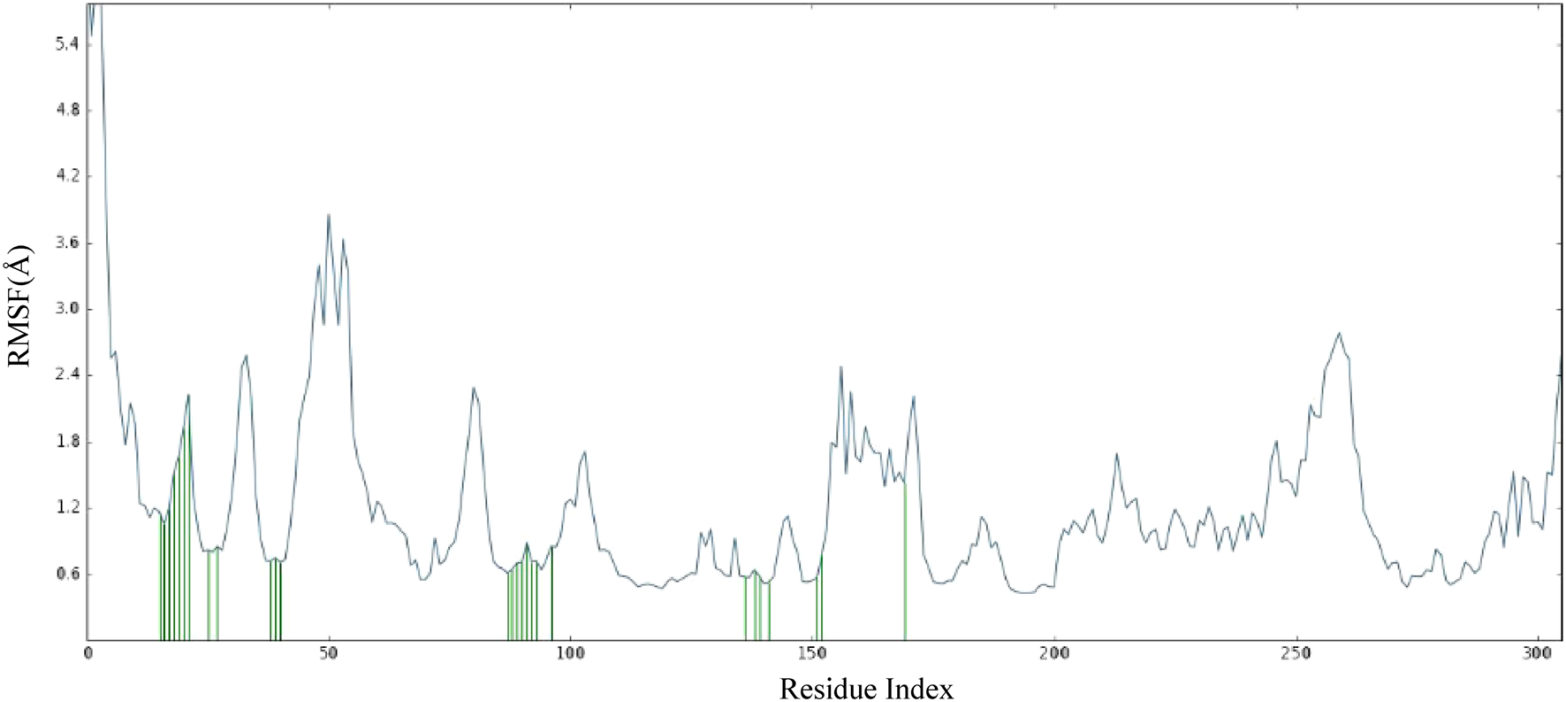
The residue wise fluctuations of CDK2 in complex with compound **3f**.

Root Mean Square Fluctuation (RMSF) serves as a metric quantifying the average deviation of each atom's position from its mean position within a specified simulation or ensemble of structures. It furnishes insights into the flexibility or mobility of the residue, with a higher RMSF value signifying increased flexibility or mobility of the residue. The results of the RMSF study inferred that the majority of amino acid residues exhibited a notable level of stability. Some residues, however, show greater RMSF values than others, suggesting that they are more flexible and have been considerably impacted. For example, amino acid 51 has the highest RMSF value in the dataset, 3.86, showing that this residue is very flexible. Also, some residues with low RMSF values are less flexible. As an example, amino acid 195 has a very low RMSF value of 0.42, which is one of the lowest values in the dataset and means that it is relatively rigid. It is noteworthy that important residues of the protein of interest consistently maintained contact with compound **3f**. Amino acid residues involved in interactions with the ligand are indicated by "green lines" in Fig. 8, while the remaining residues are depicted without any dashes. It was observed that when comparing the RMSF values of residues in contact with a ligand to those that are not in contact, no discernible pattern or trend emerged. Some residues engaged with a ligand exhibited high RMSF values, while others displayed low RMSF values. Similarly, some residues that did not come into contact with a ligand demonstrated high RMSF values, while others displayed low RMSF values.

**Figure 9:**
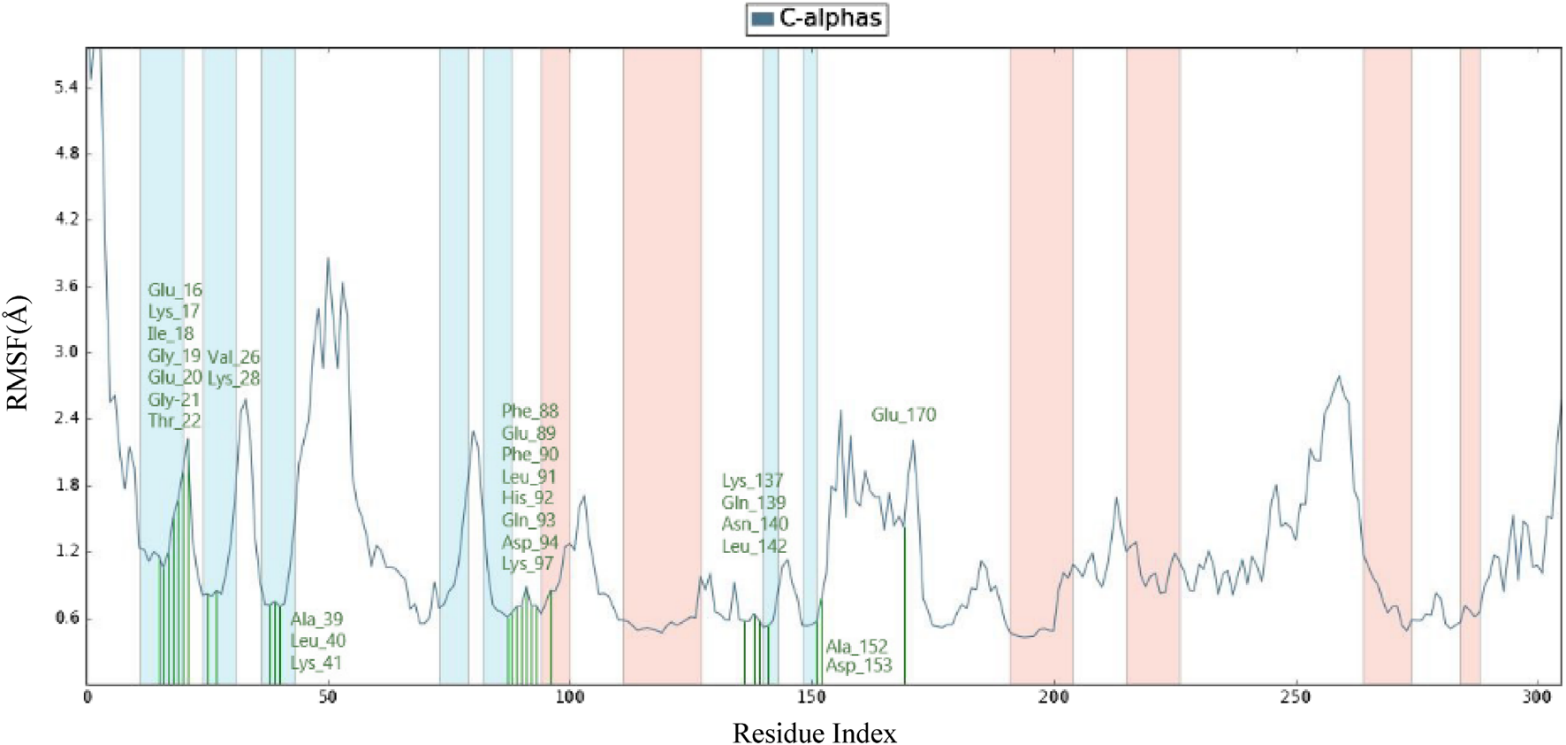
RMSF plot for Cα of CDK2 residues in compound **3f.**-CDK2 complex.

The comprehensive analysis of the interaction between compound **3f.** and the binding site residues of CDK2 is illustrated in Fig. 9. The residues that interact with compound **3f.** are as follows: Glu16, Lys17, Gly19, Val26, Glu20, Lys28, Gly21, Thr22, Al39, Leu40, Lys41, Phe88, Glu89, Phe90, Leu91, His92, Gln93, Asp94, Lys97, Lys137, Gln139, Asn140, Leu142, Al152, Asp153, and Glu170. The residue that interacts with compound **3f.** is represented by the color green. The visualization indicates that the protein's secondary structures, helices and β-strands, are represented by the orange and blue bands, respectively. The RMSF values for the residues in the binding site were found to be less than 2 Å.

**Figure 10:**
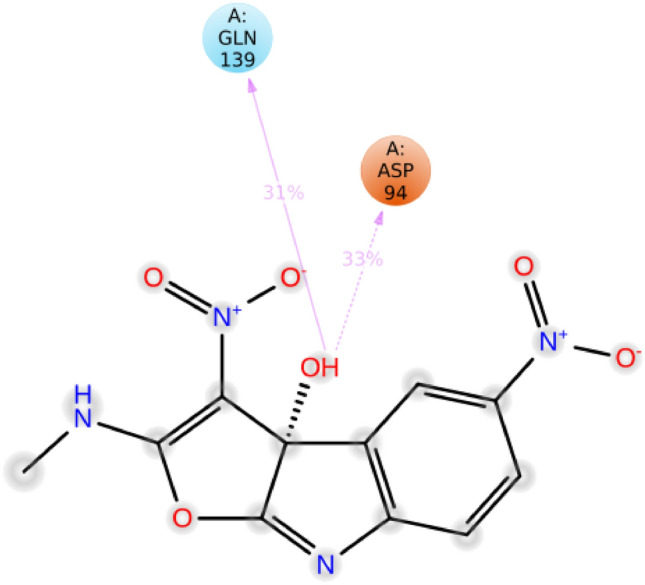
Schematic of detailed ligand atom interactions with the protein residues.

The interactions between **3f.** and the CDK2 active site pocket, which occurred for more than 30% of the simulation, are shown in Fig. 10. To summarize, the interactions may be succinctly described as follows: (1) A hydrogen bond formed between Gln139 and the hydroxyl group of the 2-(methylamino)-3, 5-dinitro-3a*H*-furo[2,3-*b*]indol-3a-ol. (2) An charge interaction took place between Asp94 and the hydroxyl group of the 2-(methylamino)-3, 5-dinitro-3a*H*-furo[2,3-*b*]indol-3a-ol molecule.

**Figure 11:**
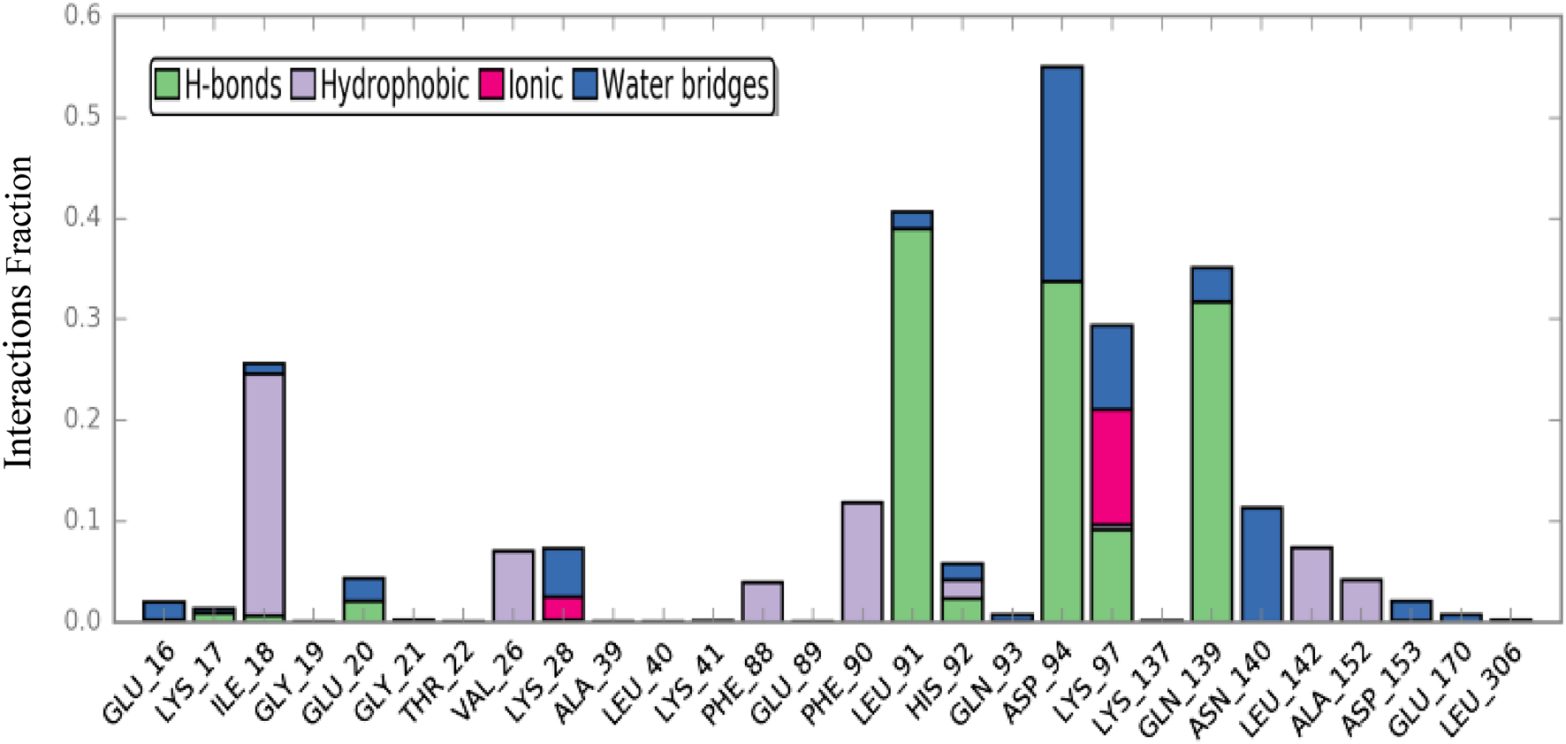
Protein–ligand contacts during simulation time.

The contributing energy components of non-covalent interactions during the simulation are then shown in Fig. 11. The X-axis delineates the interacting residues at the active site with the ligand, while the Y-axis signifies the fraction of simulation time for the interaction. The stacked bar charts are normalized over the entire trajectory. As depicted in Fig. 11, Ile18 engaged in hydrophobic interactions with the ligand for approximately 20% of the simulation duration. Moreover, over at least 35% of the simulation period, Leu91, Asp94, and Gln139 formed hydrogen-bond interactions with the ligand. Significantly, Lys97 demonstrated a variety of interactions, encompassing ionic, water-bridged, and hydrogen-bond interactions with the ligand. Consequently, this residue experienced numerous interactions throughout the entire simulation time.

**Figure 12:**
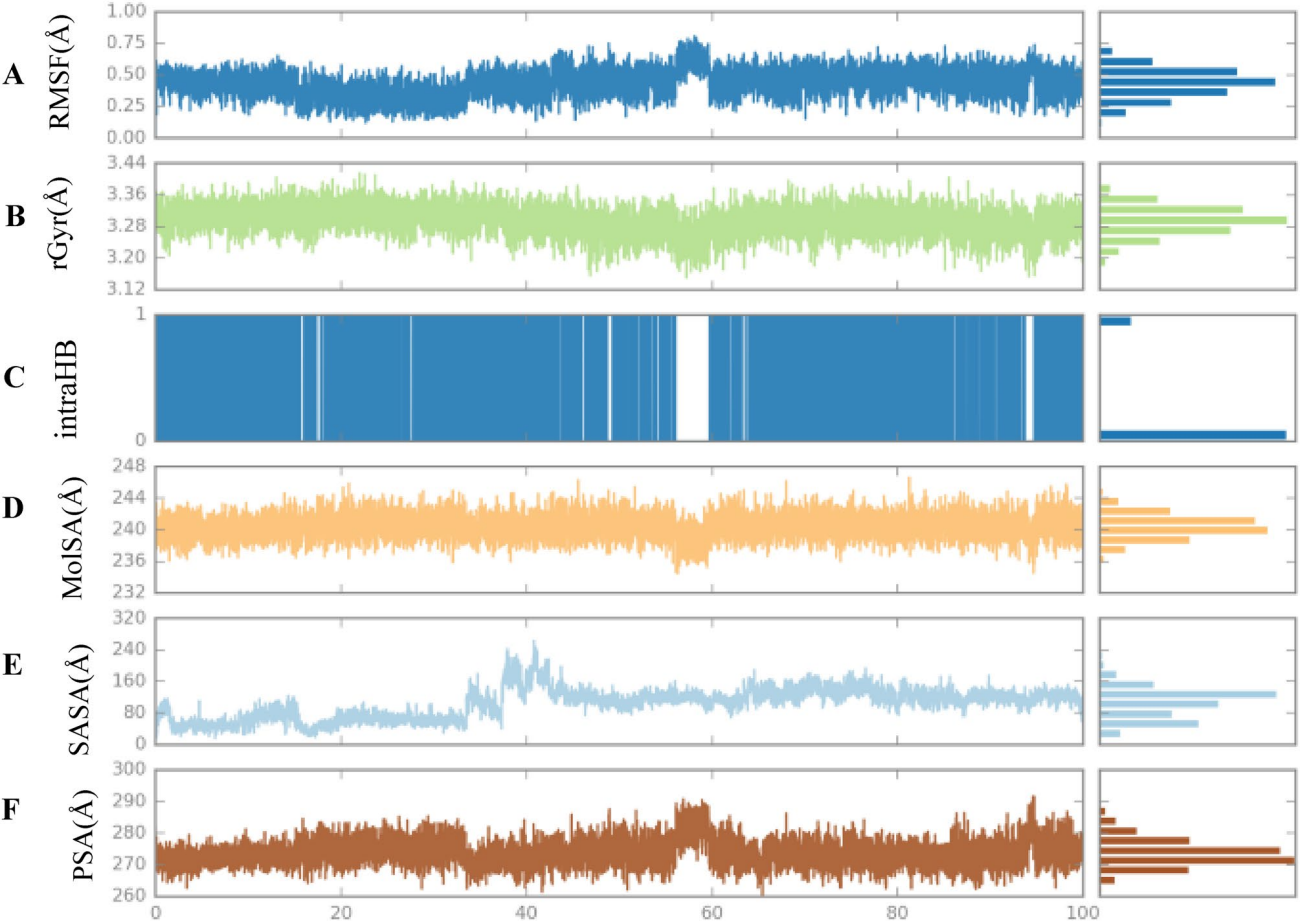
(**A**) RMSD, (**B**) rGyr, (**C**) intraHB, (**D**) MolSA, (**E**) SASA, and (**F**) PSA of the ligand–protein complex as calculated during the 100 ns of MD Simulation.

In order to determine the stability of ligand **3f.** in the CDK2 receptor throughout the 100-ns simulation depicted in Fig. 12, an examination was conducted on six parameters^20^. The maximum RMSD of **3f.** during the simulation was 0.75 Å. In the initial stage, fluctuations were observed from 0 to 20 ns, followed by a stable RMSD throughout the entire simulation process. The radius of gyration fluctuated until 100 ns, and then a stable conformation was attained over the whole simulation time. During the 100 ns simulation, the radius of gyration for compound **3f.** varied between 3.18 and 3.38 Å. Strong intramolecular H-bond interactions indicated that compound **3f.** possessed a potent inhibitory capacity. The SASA plot exhibited a variable pattern for the first 42 ns, followed by a period of stability until the simulations concluded. The MolSA and PSA plots provided evidence of the stability of ligand **3f.** throughout the simulation time.

**Figure 13:**
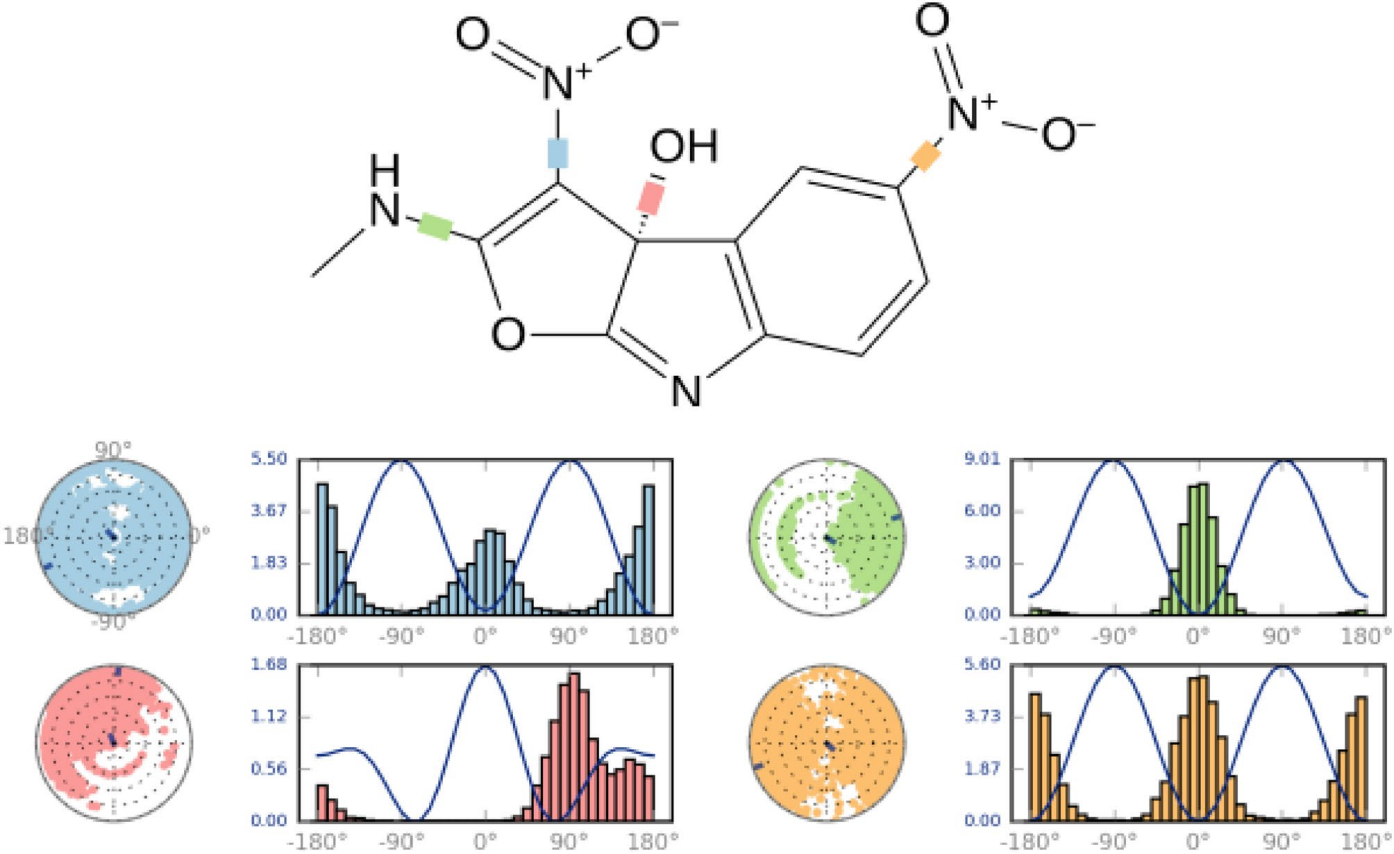
Torsional analysis of compound **3f.** conformations.

Figure 13 displays a 2D schematic of compound **3f.**, with rotatable bonds that are color-coded. The rotatable torsional bond of compound **3f.** was supplemented by a radial plot and the same color bar plots. A radial plot and the same color bar plots were used to augment compound **3f.**'s rotatable torsional bond. The time progression was represented radially outwards from the center of the radial plot, which depicts the simulation's inception. The probability density of the torsion angle was shown using bar plots, which provided a concise summary of the data presented in the radial plots. The Y-axis of the bar plots depicted the rotational bond potential, expressed in units of kcal/mol. The radial and bar plots elucidated the torsional potential interactions and the conformational strain of compound **3f.** while maintaining a conformation bound to a protein.

#### Drug-likeness prediction

Drug likeness is the degree to which certain compounds and well-known drugs are similar to each other. The foundation of this phenomenon rests on a delicate equilibrium between molecular and structural characteristics. The assessment of drug-likeness includes the evaluation of several molecular attributes, such as hydrophobicity, electronic distribution, hydrogen bonding, molecular weight, pharmacophore entity, bioavailability, reactivity, toxicity, and metabolic stability^21^. Lipinski's rule is a commonly employed approach in the assessment of the solubility and permeability characteristics of compounds, enabling the prediction of their viability as prospective drug candidates. Based on this principle, it may be inferred that compounds that contravene Lipinski's rule of five are more likely to manifest inadequate absorption or penetration. The derivatives were thoroughly examined using the SwissADME online web server^22^. None of the compounds **3a–f** examined in this study violate the Lipinski rule, as their values fall within the acceptable range and demonstrate satisfactory absorption properties. Moreover, it is noteworthy that these compounds **3a–f** occupy a favorable region within the physiochemical space, hence justifying their classification as potential lead compounds. The pharmacokinetic analysis demonstrated that the investigated compounds, namely **3a–e**, exhibit favorable absorption characteristics inside the gastrointestinal system following oral administration. Additionally, these compounds were found to be susceptible to efflux by P-glycoprotein (P-gp). Conversely, compound **3f.** had limited absorption via the gastrointestinal tract. This observation can be attributed to the higher molar refractivity (MR) value of **3f.** in comparison to the MR values of other compounds that were examined. The application of pan-assay interference substances (PAINS) structural warnings has been employed in the field of pharmaceutical chemistry to identify regions within a compound's structure that are prone to instability, reactivity, and toxicity^23,24^. None of the compounds **3a–f** exhibit any alarms in the PAINS descriptions, indicating their potential as promising therapeutic candidates. The synthetic accessibility score (SA score) is a quantitative measure employed to assess the level of difficulty associated with synthesizing drug-like compounds. It was noted that all of the compounds have a favorable SA, suggesting their potential for facile synthesis (Table 5).

**Table 5:** Physicochemical, pharmacokinetics, and medicinal chemistry properties of the compounds **3a–f**.

	MW (g/mol)	HBA	HBD	TPSA (Å^2^)	Consensus Log Po/w *	MR	GI Absorption	BBB Permeant	P-gp Substrate	Lipinski	Pfizer	PAINS (alert)	Bioavailability Score	Synthetic accessibility score
**3a**	326.10	5	2	99.67	1.16	74.40	High	No	No	Yes	Yes	0	0.55	3.96
**3b**	281.65	5	2	99.67	1.00	71.71	High	No	No	Yes	Yes	0	0.55	3.91
**3c**	265.20	6	2	99.67	0.81	66.66	High	No	No	Yes	Yes	0	0.55	3.93
**3d**	281.65	5	2	99.67	0.96	71.71	High	No	No	Yes	Yes	0	0.55	3.94
**3e**	247.21	5	2	99.67	0.43	66.70	High	No	No	Yes	Yes	0	0.55	3.95
**3f.**	292.20	7	2	145.49	− 0.30	75.52	Low	No	No	Yes	Yes	0	0.55	4.03

MW: Molecular Weight; HBA: Num. H-Bond Acceptors; HBD: Num. H-Bond Donors; NRB: Number of rotatable bonds; MR: Molar Refractivity; TPSA: Topological Polar Surface Area; P-M: Poor-Moderate; P: Poor; GI: Gastrointestinal; P-gp, P Glycoprotein;* Average of five prediction.

#### ADMET properties

In the process of advancing therapeutic drug development, a profound understanding of pharmacology and toxicology is crucial. The acquisition of this knowledge not only serves to reduce the period of medication development but also augments the success rate. ADMET indices, comprising Absorption, Distribution, Metabolism, Excretion, and Toxicity, are frequently utilized to assess the characteristics of a compound. The parameters for furo[2,3-*b*]indol-3a-ol derivatives are obtained using the online web server ADMET Lab 2.0^25^. The utilization of CaCo-2 cells, which are generated from human colon epithelial cells, is a prevalent approach for assessing the absorption of pharmaceutical substances within the human digestive tract. On the other hand, Madin Darby Canine Kidney (MDCK) cells are of particular value in evaluating the swift permeability of drug molecules, as they possess a shorter growing time in comparison to CaCo-2 cells^26^. The CaCo-2 cell permeability data obtained for the synthesized compounds demonstrated values that fell within a satisfactory range, hence indicating favorable membrane permeability characteristics for these compounds. All furo[2,3-*b*]indol-3a-ol derivatives exhibited favorable MDCK cell permeability, suggesting a heightened likelihood of renal cell-mediated removal. In terms of Plasma glycoprotein (PGP) inhibitors and PGP substrates, all compounds were shown to be PGP inhibitors and substrates. The computed values for human intestinal absorption (HIA) indicate that all substances possess a high likelihood of being effectively absorbed through the intestinal membrane. The assessment of plasma protein binding (PPB) is a crucial determinant in evaluating the safety profile of medications. Drugs with a high PPB value (> 90%) often exhibit a narrow therapeutic index, indicating a smaller margin of safety. Conversely, pharmaceuticals with a low PPB value are generally considered to be safer. In the current study, it was shown that all compounds **3a–f** had low plasma protein binding (PPB) values, indicating a wide therapeutic index for these compounds. Compounds that have CBrain/CBlood values greater than 1 are categorized as possessing central nervous system (CNS) activity, whereas compounds with CBrain/CBlood values below 1 are characterized as lacking CNS activity. Compounds exhibiting central nervous system (CNS) activity demonstrate the capacity to traverse the Blood–Brain Barrier (BBB) and induce adverse effects on the central nervous system^27^. Based on the data provided in Table 6, it can be seen that the CBrain/CBlood values of all the compounds are less than 1, suggesting their inability to traverse the Blood–Brain Barrier (BBB). As a result, the compounds we have synthesized exhibit a lack of neurotoxicity.

**Table 6:** ADMET profile of the compounds **3a–f**.

Metabolism											Elimination	
	CYP1A2 inhibitor	CYP1A2 Substrate	CYP2C19 inhibitor	CYP2C19 Substrate	CYP2C9 inhibitor	CYP2C9 Substrate	CYP2D6 inhibitor	CYP2D6 Substrate	CYP3A4 inhibitor	CYP3A4 Substrate	T_1/2_	CL
**3a**	+ + 0.861	-0.324	+ + 0.861	+ + 0.815	− 0.493	–-0.058	–-0.009	–0.289	+ 0.676	+ + 0.837	0.604	2.757
**3b**	+ + 0.838	-0.406	+ + 0.802	+ + 0.814	− 0.419	–-0.057	–-0.010	–0.281	+ 0.574	+ + 0.880	0.574	4.756
**3c**	+ 0.661	–0.231	+ 0.666	+ + 0.781	–0.266	–-0.058	–-0.005	-0.354	-0.331	+ + 0.793	0.434	4.992
**3d**	+ 0.601	–0.210	+ + 0.765	+ + 0.841	− 0.421	–-0.064	–-0.009	–0.182	+ 0.693	+ + + 0.905	0.557	4.825
**3e**	-0.478	–0.113	+ 0.542	+ + 0.812	–0.227	–-0.063	–-0.003	–0.254	-0.327	+ + 0.869	0.600	4.489
**3f.**	+ + 0.737	–0.214	+ 0.685	+ 0.580	− 0.340	–-0.061	–-0.004	–0.232	-0.340	+ + 0.722	0.512	4.402

Absorption and Distribution												
	Caco-2 Permeability	MDCK Permeability	PGP-Inhibitor	*p*-Glycoprotein substrate (PGPsubstrate)	Human intestinal absorption (HIA)	Plasma protein binding (PPB)	Volume of distribution (VD)	Blood brain barrier (BBB) penetration (c.brain/ c.blood)				
**3a**	− 4.945	0.00031	0	–-0.025	– –0.095	59.31	0.935	+ + 0.808				
**3b**	− 4.968	0.00021	0	–0.234	– –0.006	71.31	0.992	+ + 0.898				
**3c**	− 5.017	0.00025	0	–0.193	– –0.007	55.26	0.949	+ + 0.741				
**3d**	− 4.883	0.00018	0	–0.138	– –0.008	61.91	1.163	+ + 0.875				
**3e**	− 5.005	0.00026	0	–0.293	– –0.006	41.80	0.907	+ + 0.821				
**3f.**	− 5.093	0.00041	0	+ 0.518	– –0.007	66.07	0.914	− 0.414				

Toxicity												
	AMES toxicity	Carcinogenicity	Eye corrosion	Eye irritation	hERG	H-HT	LD_50_	Respiratory toxicity				
**3a**	− 0.326	+ + 0.823	– –0.004	– –0.082	– –0.008	+ + 0.853	1751.293	+ + + 0.945				
**3b**	+ + 0.808	+ + 0.728	– –0.003	– –0.034	– –0.012	+ + + 0.925	1261.007	+ + + 0.939				
**3c**	+ + + 0.906	+ + 0.828	– –0.003	– –0.037	– –0.010	+ + + 0.954	838.636	+ + + 0.939				
**3d**	+ + 0.738	+ + 0.730	– –0.003	– –0.042	– –0.009	+ + 0.865	1302.319	+ + + 0.955				
**3e**	+ + 0.803	+ 0.633	– –0.004	– –0.100	– –0.006	+ + + 0.925	1122.189	+ + + 0.933				
**3f.**	+ + + 0.968	+ + 0.791	– –0.003	–0.122	– –0.010	+ + + 0.968	1785.215	+ + + 0.921				

For the classification endpoints, the prediction probability values are transformed into six symbols: 0–0.1(---), 0.1–0.3(--), 0.3–0.5(-), 0.5–0.7(+), 0.7–0.9(++), and 0.9–1.0(+++).

## Conclusion

In the present investigation, a series of furo[2,3-*b*]indol-3a-ol derivatives were synthesized and then subjected to analysis using IR, Mass, ^1^H, and ^13^C NMR. The use of DFT calculations proved effective in the accurate prediction of structural geometry. Furthermore, molecular docking was performed on all of the compounds under consideration. The results indicated that all compounds had binding affinity with the CDK2 protein. Significantly, out of these compounds, **3f.** demonstrated the most elevated binding energy values. The ligand–protein complex underwent MD simulation to assess stability, RMSD, and RMSF values. In addition, in *silico* ADMET studies predicted favorable drug-likeness properties for the synthesized derivatives. This comprehensive exploration offers a promising avenue for further investigation into their efficacy as CDK2 inhibitors in the context of drug development. Further validation of the chemoinformatics study's conclusions would need more in vivo and in vitro investigations. Further experimentation using in *vitro* and in *vivo* studies would be necessary to validate the findings of the chemoinformatics investigation.

### Experimental

#### General information

All solvents and reagents were purchased from Aldrich and Merck Chemical Co. DMSO-*d*_*6*_ and acetone-*d*_*6*_ solvents were used to obtain NMR spectra on a Bruker (400 MHz for ^1^H and 100 MHz for ^13^C). Melting points were measured using an electrothermal 9100. An Agilent 5975C VL MSD with a Triple-Axis detector recorded mass spectra at 70 eV. IR spectra were measured using the Bruker Tensor 27.

#### General method for synthesizing compounds 3a–f

A mixture of *N*-methyl-1-(methylthio)-2-nitroethenamine (0.5 mmol), isatin derivatives (0.5 mmol), and sulfamic acid (0.05 mmol) were magnetically stirred in EtOH/H_2_O (1:3, 4.0 mL) at reflux for 24 h. TLC was used to monitor the reaction, and the eluent used was a 1:1 ratio of ethyl acetate to *n*-hexane. Following the conclusion of the reaction, compounds **3a–f** were obtained by filtering and washing the precipitated product with EtOH. The reaction was TLC-monitored. After the reaction, the precipitate was filtered and washed with EtOH to yield compound **3a-f**.

#### 5-Bromo-2-(methylamino)-3-nitro-3a*H*-furo[2,3-*b*]indol-3a-ol (3a)

White solid; yield: 90%; mp 367–368 °C. IR (KBr): 3434 (OH), 3272 (NH), 1693 (C=N), 1260 (C–N) 1534 and 1376 cm^-1^ (NO_2_).^1^H NMR (400 MHz, DMSO-*d*_*6*_): δ ppm, 13.05 (1H, *s*, OH), 9.07 (1H,*br q*, ^3^*J*=4.52 Hz, NH), 7.92 (1H, *dd*, ^3^*J*=8.8 Hz, ArH), 7.72 (1H, *d*, ^4^*J*=1.6 Hz, ArH), 7.41(1H, *d*, ^3^*J*=8.8 Hz, ArH), 2.83 (3H, *d*, ^3^*J*=4.5 Hz, CH_3_). ^13^C NMR (100 MHz, DMSO-*d*_*6*_): δ ppm, 26.5 (N–CH_3_), 115.8 (C–OH), 116.2 (C-NO_2_), 119.0 (C–Br), 129.5, 136.7, 134.8, 139.2, 139.9 (Ar), 153.9 (C=N),161.3 (C–N). MS (EI, 70 eV): *m/z* (%)=324 (0) [M] ^+^, 301.1 (23), 227 (41), 225 (42), 199 (98), 197 (100), 171 (25), 170 (24), 169 (24), 149 (24), 90.1 (27), 63.1(39).

#### 5-Chloro-2-(methylamino)-3-nitro-3a*H*-furo[2,3-*b*]indol-3a-ol (3b)

White solid; yield: 80%; mp 367–368 °C. IR (KBr): 3456 (OH), 3275 (NH), 1683 (C=N), 1264(C–N) 1528 and 1365 cm^-1^ (NO_2_). ^1^H NMR (400 MHz, DMSO-*d*_*6*_): δ ppm, 11.69 (1H, *br s*, OH), 8.80 (1H, *br s*, NH), 7.77–7.73 (2H, *m,* ArH), 7.59 (1H, *d*, ^3^*J*=8.8 Hz, ArH), 2.99 (3H, *d*, ^3^*J*=4.6 Hz, CH_3_). ^13^C NMR (100MHz, DMSO-*d*_*6*_): δppm,26.0(N–CH_3_),115.8(C–OH),118.3(C-NO_2_),126.1(Ar), 127.6(Ar),133.6(C–Cl),137.6,138.8,139.5(Ar),153.4(C=N),160.8 (C-N,). MS (EI, 70 eV): *m/z* (%)=281.1 (100) [M] ^+^, 251.1 (27), 207.1 (32), 180.1(26), 178.1 (49), 177.1 (42), 150.1 (42), 114.1 (52), 87.1 (22), 58.2 (39).

#### 5-Fluoro-2-(methylamino)-3-nitro-3a*H*-furo[2,3-*b*]indol-3a-ol (3c)

White solid; yield: 72%; mp 354–356 °C. IR (KBr): 3434 (OH), 3264 (NH), 1650 (C=N), 1246(C–N) 1542 and 1367 cm^−1^ (NO_2_). ^1^H NMR (400 MHz, DMSO-*d*_*6*_): δ ppm, 13.00 (1H, *br s*, OH), 9.05 (1H, *br q*, ^3^*J*=4.6 Hz, NH), 7.75–7.64 (1H, *m*, ArH), 7.54–7.46 (1H, *m*, ArH), 7.42 (1H, *dd*, ^3^*J*=9.2 Hz, ArH), 2.82 (3H, *d*, ^3^*J*=4.64 Hz, CH_3_). ^13^C NMR (100 MHz, DMSO-*d*_*6*_): δ ppm, 25.9 (N-CH_3_), 112.3 (C–OH), 114.7(C–NO_2_), 118.5, 122.0, 122.3, 135.7, 153.3(Ar), 156.4(C-–F), 158.8(C=N), 160.9 (C–N). MS (EI, 70 eV): *m/z* (%)=265 (18) [M]^+^, 247.1 (100), 217 (34), 173 (41), 144 (45), 143.1 (24), 116.1 (34), 115.1 (39), 89.1 (24), 58.1 (27).

#### 7-Chloro-2-(methylamino)-3-nitro-3a*H*-furo[2,3-*b*]indol-3a-ol (3d)

White solid; yield: 50%; mp 322–324 °C. IR (KBr): 3456 (OH), 3264 (NH), 1683(C=N), 1212 (C-N) 1550 and 1360 cm^−1^ (NO_2_). ^1^H NMR (400 MHz, DMSO-*d*_*6*_): δ ppm, 12.34 (1H, *br s*, OH), 9.08 (1H,*br q*, ^3^*J*=4.56 Hz, NH), 7.93 (1H, *dd*, ^3^*J*=8.4 Hz, ArH), 7.63 (1H, *dd*, ^3^*J*=8.4 Hz, ArH), 7.39 (1H, *t*, ^3^*J*=8.0 Hz, ArH), 2.82(3H, *d*, ^3^*J*=4.64 Hz, CH_3_). ^13^C NMR (100 MHz, DMSO-*d*_*6*_): δ ppm, 25.9 (N–CH_3_), 116.7(C–OH), 119.6(C-NO_2_), 124.9(Ar), 127.2(C–Cl), 133.4, 140.6, 140.0(Ar), 154.1 (C=N), 161.3(C-N). MS (EI, 70 eV): *m/z* (%)=281.1 (100) [M] ^+^, 251 (23), 207 (40), 180 (18), 178.1 (48), 177.1 (24), 150.1 (27), 114.1 (43), 87.1 (13), 58.1 (23).

#### 2-(methylamino)-3-nitro-3a*H*-furo[2,3-*b*]indol-3a-ol(3e)

White solid; yield: 70%; mp 337–338 °C. IR (KBr): 3448 (OH), 3279 (NH), 1646 (C=N), 1267 (C–N) 1365 and 1542 cm^−1^ (NO_2_). ^1^H NMR (400 MHz, DMSO-*d*_*6*_): δ ppm, 11.58 (1H, *br s*, OH), 9.07 (1H, *br s*, NH), 7.78–7.75 (2H, *m,* ArH), 7.58–7.55 (1H, *m*, ArH), 7.42–7.38(1H, *m,* ArH), 2.99 (3H, *d*, ^3^*J*=4.76 Hz, CH_3_). ^13^C NMR (100 MHz, DMSO-*d*_*6*_): δ ppm, 25.6 (N-CH_3_), 112.2(C–OH), 116.0(C–NO_2_), 123.6, 127.8, 133.3, 138.3, 139.1, 140.5(Ar), 153.6 (C=N), 161.7(C-N). MS (EI, 70 eV): *m/z* (%)=247.1 (100) [M] ^+^, 217.1 (33), 173.1 (41), 149 (55), 144.1 (44), 143.1 (23), 116.1 (32), 115.1 (38), 89.1 (23), 58.1 (25).

#### 2-(methylamino)-3, 5-dinitro-3a*H*-furo[2,3-*b*]indol-3a-ol (3f.)

Red solid; yield: 60%; mp 364–366 °C. ^1^H NMR (400 MHz, DMSO-*d*_*6*_): δ ppm, 13.46 (1H, *br s*, OH), 9.15 (1H,*br q*, ^3^*J*=4.64 Hz, NH), 8.55 (1H, *dd*, ^3^*J*=9.6 Hz, ArH), 8.37 (1H, *d*, ^3^*J*=4.0 Hz, ArH), 7.62 (1H, *d*, ^3^*J*=8.0 Hz, ArH), 2.86 (3H, *d*, ^3^*J*=4.64 Hz, CH_3_). ^13^C NMR (100 MHz, DMSO-*d*_*6*_): δ ppm, 26.0 (N-CH_3_), 113.7(C–OH), 117.7(C-NO_2_), 123.4, 127.8, 139.2, 139.1, 140.0(Ar), 142.8(C–NO_2_), 153.8 (C=N), 161.5(C-N).

### Computational studies

#### Study on a computer quantum chemical calculation based on DFT

DFT estimates molecular electron density and energy well. It determines atom, molecule, crystal, and surface structure and interactions. The calculations were performed using the Gaussian 09W program^14^. The vibration wavenumbers were predicted employing the B3LYP method with a 6-31++G(d,p) basis set. The B3LYP functional is useful for representing harmonic vibrational numbers in small to medium-sized molecules. This method estimates positive IR frequencies, indicating that the optimized structure is at a possible energy surface minimum. Also, GuassView 6.0 was used to check check files.

#### Molecular docking studies

The CDK2 enzyme interaction mode with synthesized compounds were investigated by employing Schrodinger's Maestro Molecular Modeling platform. The protein 3D structure has been obtained from PDB (6GUH)^18^. All protein preparation for docking was done. Preparing protein using the Protein Preparation Wizard^28^ was done, and missing residues were modified. GuassView 6.0 was used to depict the synthesized compounds' structures and convert them to.pdb files for the ligprep module. Ligand was prepared using the OPLS_2005 forcefield at pH 7.0 ± 2^29^. Each binding site has a 26-A grid box created using glide with standard accuracy and flexible ligand sampling, reporting 10 poses per ligand.

#### Molecular dynamic simulation

MD simulation was conducted utilizing Desmond through the Schrödinger Maestro interface^30^. The results pertain to the MD simulation executed on the complex subsequent to the preceding docking stage. A cell was characterized as orthorhombic and filled with water molecules, representing the SPC model. sufficient Cl ions were added to the system in order to counterbalance the overall charge of the complex (Supplementary file). The duration of the simulation was 100 ns. The NPT ensemble was utilized, maintaining a constant number of atoms, a pressure of 1.01325 bar, and a temperature of 300 K. The default thermostat was the 1.0‐ps interval Nose–Hoover chain method, and the default barostat was the 2.0‐ps interval Martyna-Tobias-Klein. The maestro simulation interaction diagram was used to assess the molecular dynamic simulation.

### Supplementary Information

Supplementary Information.

## Data Availability

All data generated or analyzed during this study are included in this published article and its supplementary information files.
