# Supplementary material for: Synthesis, docking, MD simulation, ADMET, drug likeness, and DFT studies of novel furo[2,3-b]indol-3a-ol as promising Cyclin-dependent kinase 2 inhibitors
Source: Sci Rep. 2024 Feb 7;14:3084. doi: 10.1038/s41598-024-53514-1 (PMC10847505; doi:10.1038/s41598-024-53514-1)
Supplement: Supplementary file 1 — Supplementary Information. [file 41598_2024_53514_MOESM1_ESM.docx]

**Supporting Information**

**Synthesis, Docking, MD Simulation, ADMET, Drug Likeness, and DFT Studies of Novel Furo [2,3-*b*] indol-3a-ol as promising Cyclin-dependent kinase 2 inhibitors**

Davood Gheidari *^a^ Morteza Mehrdad ^a^ Mohammad Bayat *^b^

*^a^Department of Chemistry, Faculty of Science, University of Guilan, Rasht, Iran*

*^b^Department of Chemistry, Faculty of Science, Imam Khomeini International University, Qazvin, Iran*

**The Table of Contents**

| **Title** | **Page** |
| --- | --- |
| Title, author’s name, address and table of contents | 1 |
| Experimental Section; General remarks | 2 |
| **Figure. 1** Molecular structures of products **3a–f**. | 2 |
| **Figure 2**. Structure of CDK2. | 3 |
| ^1^H and ^13^C NMR and IR and Mass spectrums of **3a** | 4-5 |
| ^1^H and ^13^C NMR and IR and Mass spectrums of **3b** | 6-7 |
| ^1^H and ^13^C NMR and IR and Mass spectrums of **3c** | 8-9 |
| ^1^H and ^13^C NMR and IR and Mass spectrums of **3d** | 10-11 |
| ^1^H and ^13^C NMR and IR spectrums of **3e** | 12-13 |
| ^1^H and ^13^C NMR spectrums of **3f** | 14 |

**Experimental Section**

**General remarks:**

Melting points were measured on an Electrothermal 9100 apparatus. Mass spectra were recorded with an Agilent 5975C VL MSD with Triple-Axis Detector operating at an ionization potential of 70 Ev. ^1^H and ^13^C NMR spectra were measured (DMSO) and (acetone) with a Bruker Bio Spin spectrometer at 400 and 100 MHz, respectively. IR spectra were recorded on a Bruker Tensor 27, ῡ in cm^-1^. All NMR spectra at room temperature were determined in DMSO-*d*_6_ and acetone-*d*_6_. Chemical shifts are reported in parts per million (*δ*) downfield from an internal tetramethylsilane reference. Coupling constants (*J* values) are reported in hertz (Hz), and spin multiplicities are indicated by the following symbols: s (singlet), d (doublet), t (triplet), q (quartet), m (multiplet). All chemicals were purchased from Merck or Aldrich and were used without further purification.

* Signals related to residual ethanol, are indicated on the spectra.

|  |  |  |
| --- | --- | --- |
|  |  |  |

**Figure 1.** Molecular structures and percent yields of final compounds **3a–f**.


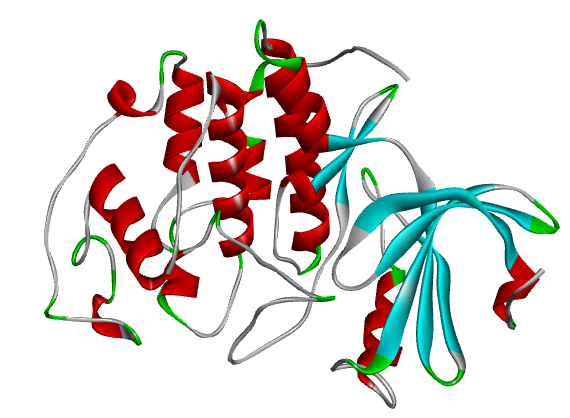


**Figure 2**. Structure of CDK2.

**
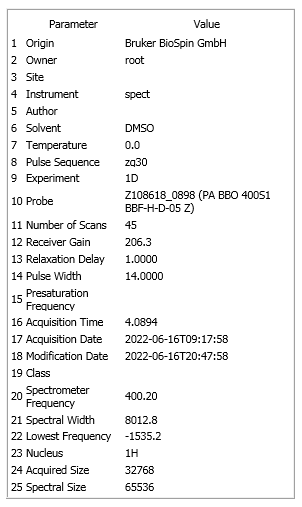
**
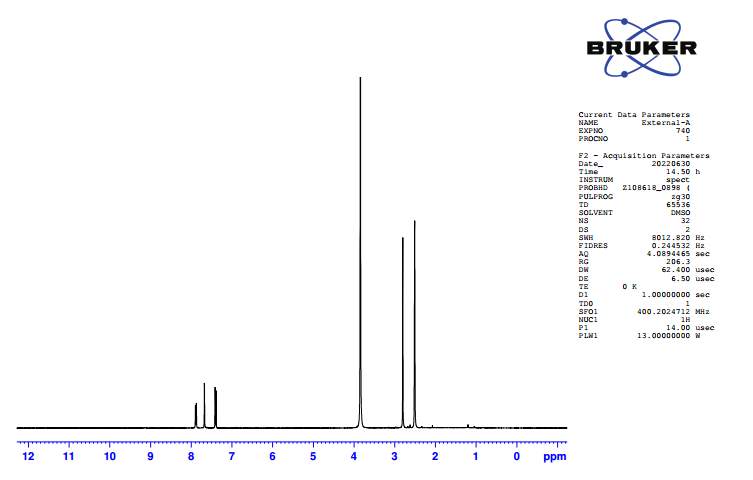


**^1^H NMR with D_2_O**

**
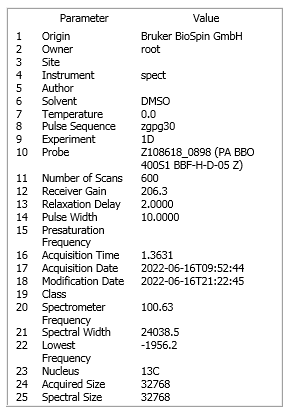
**

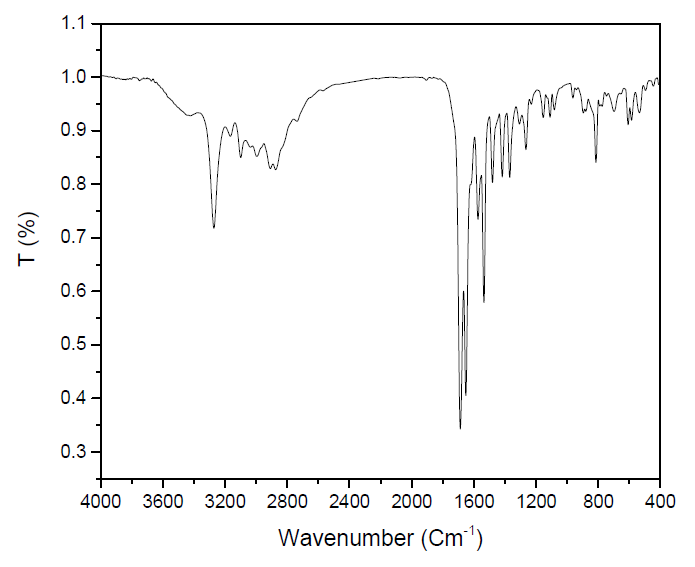


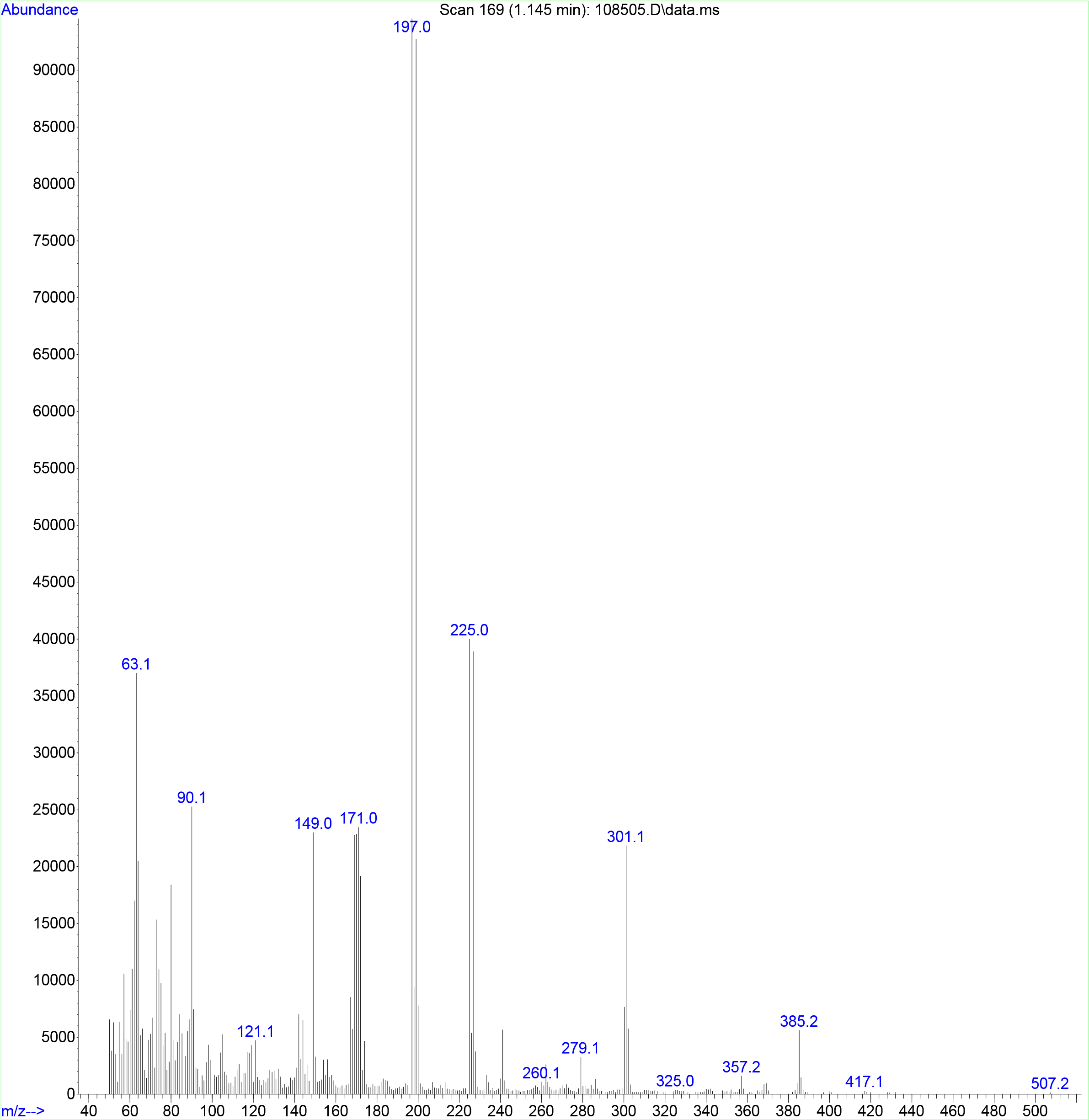


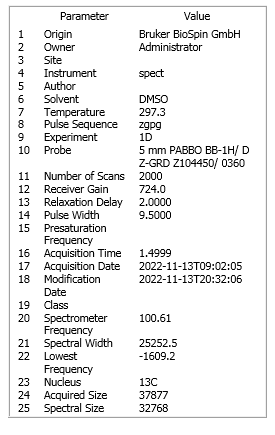


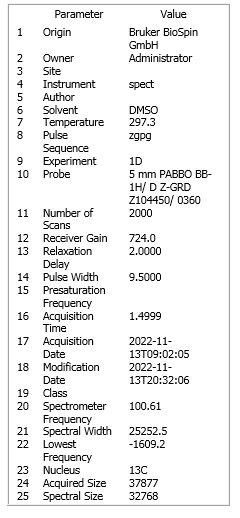


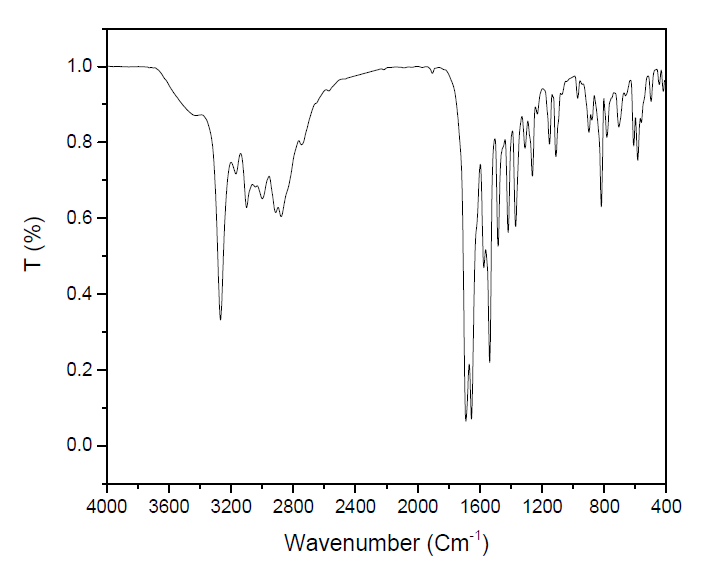


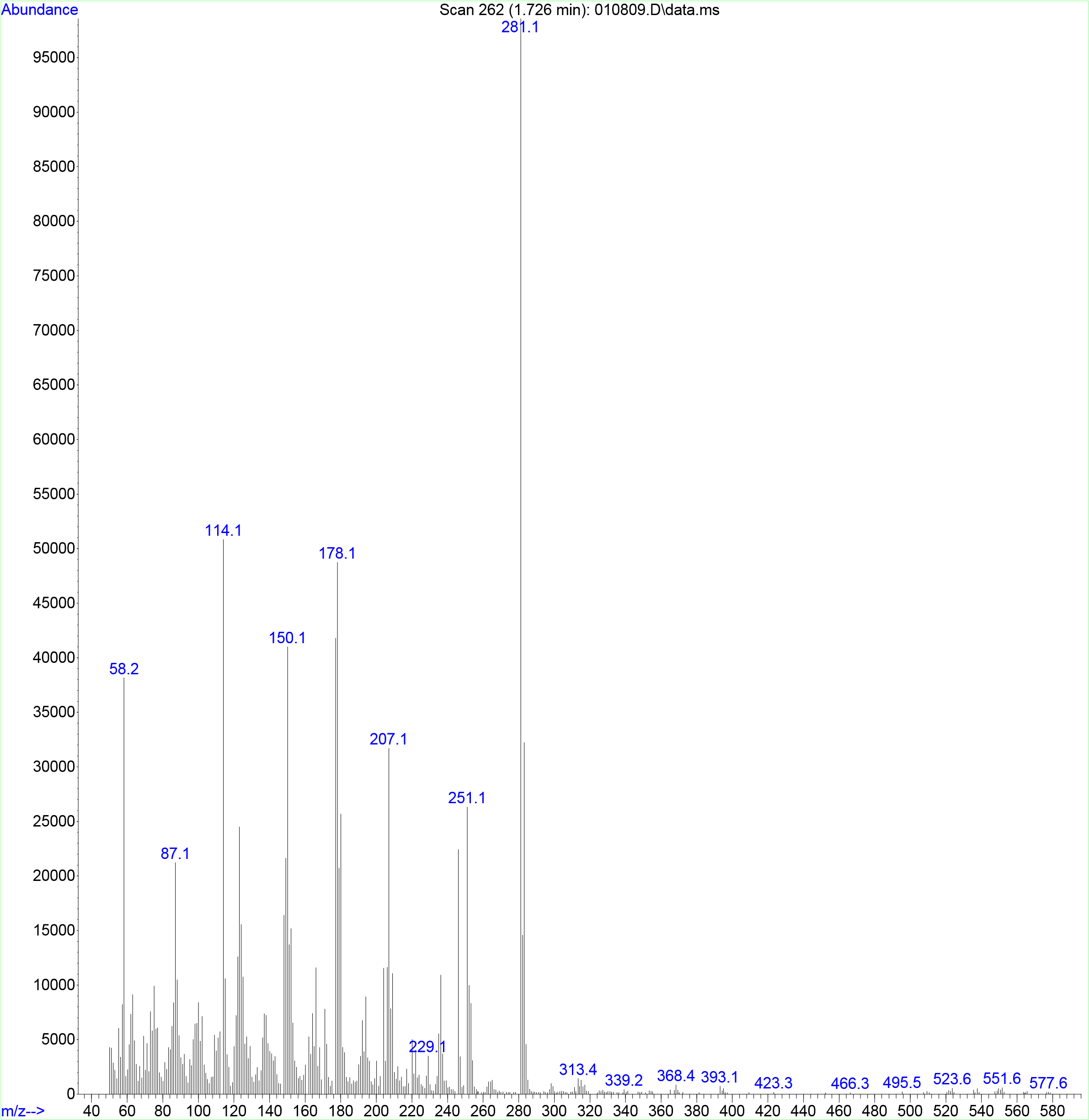


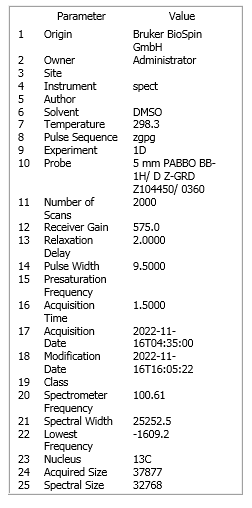


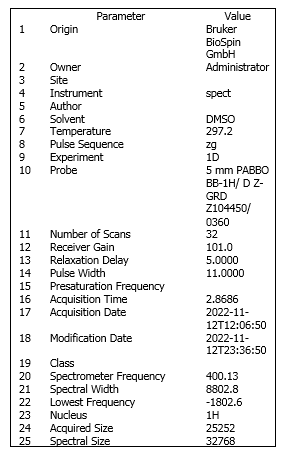


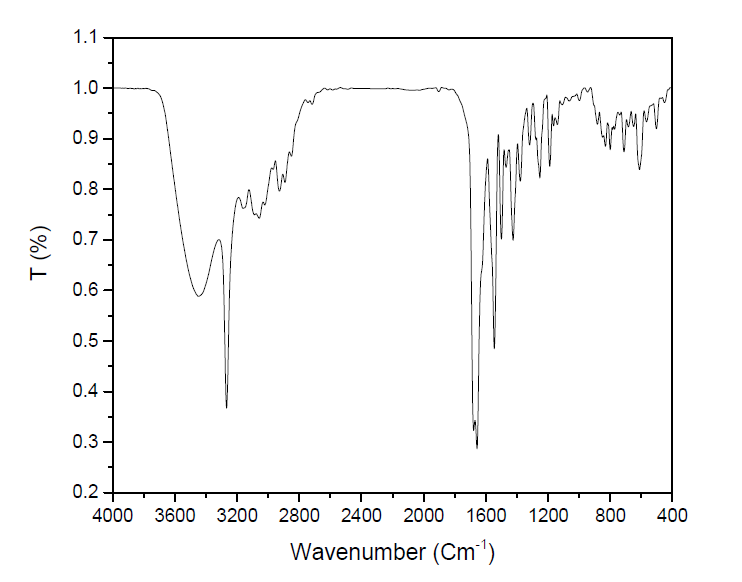


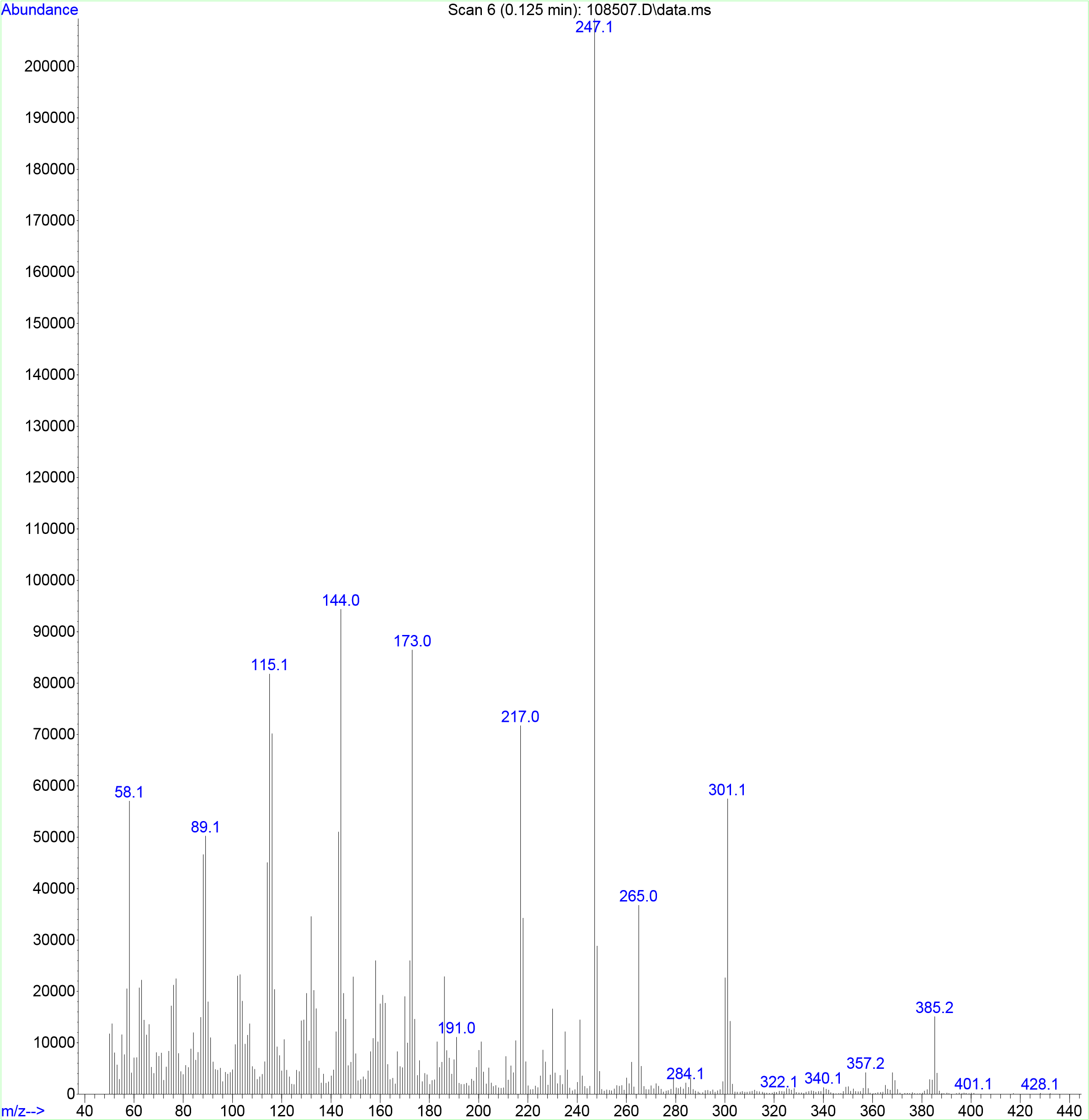


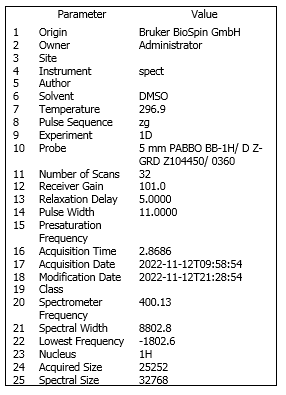


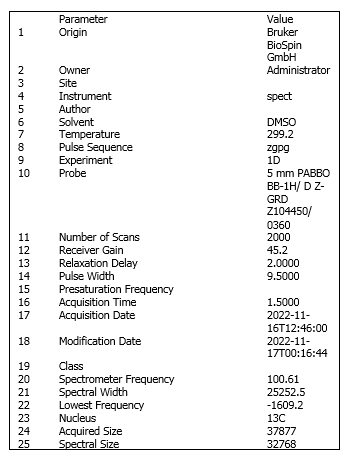


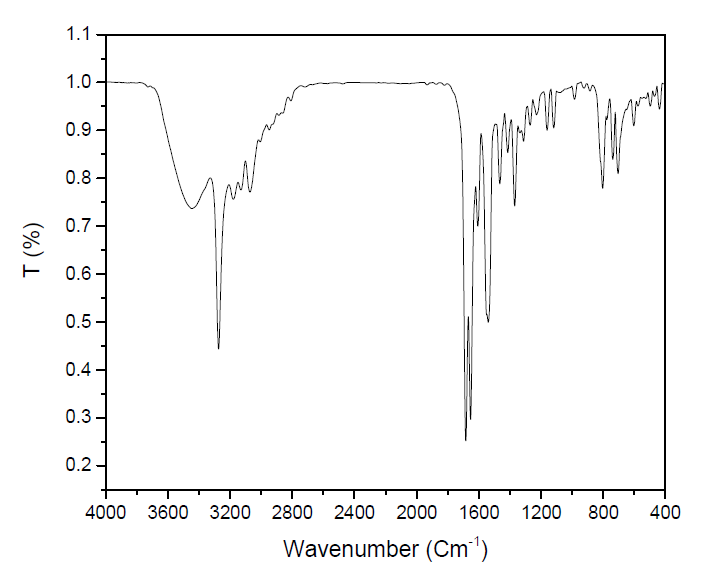


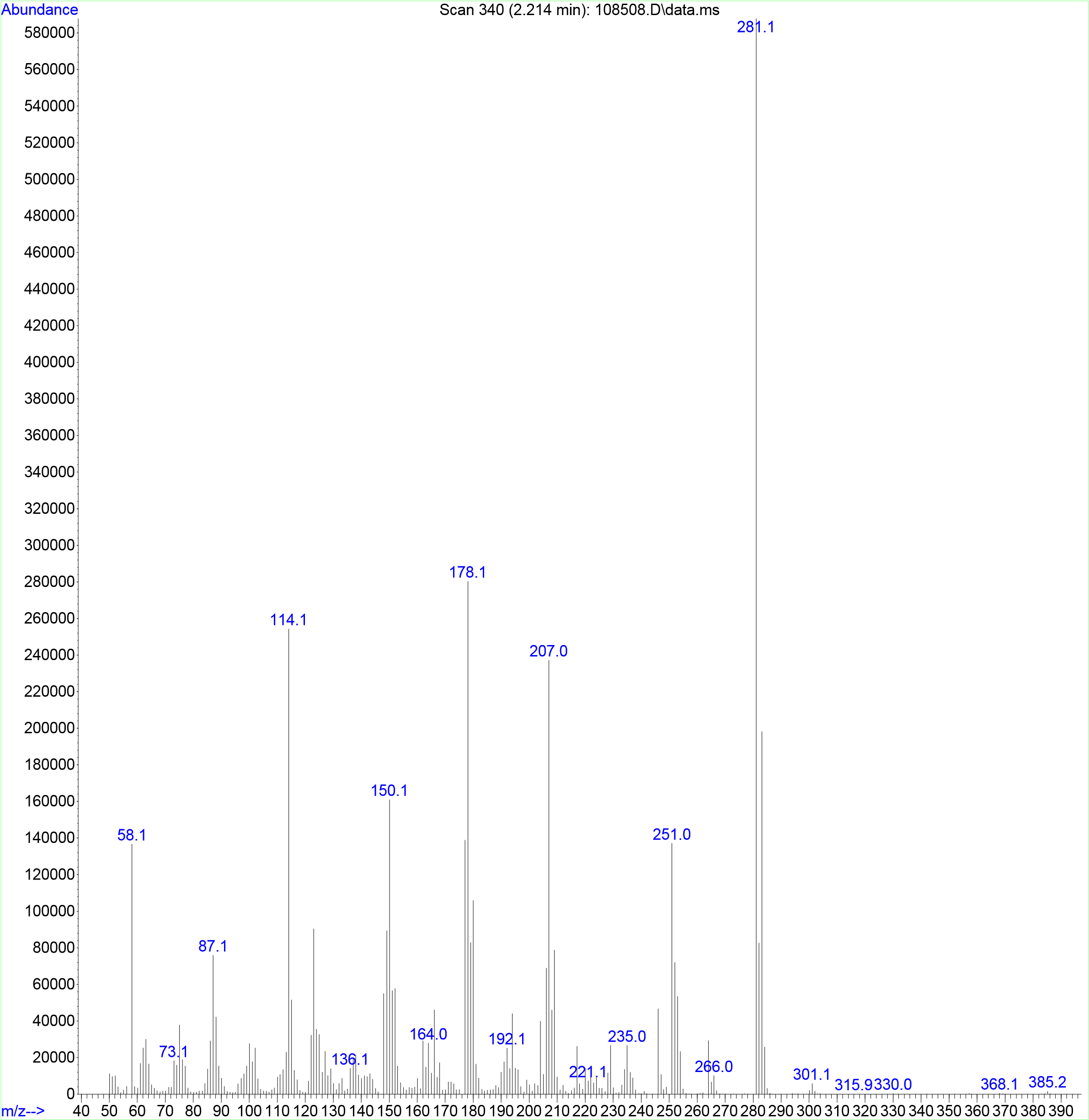


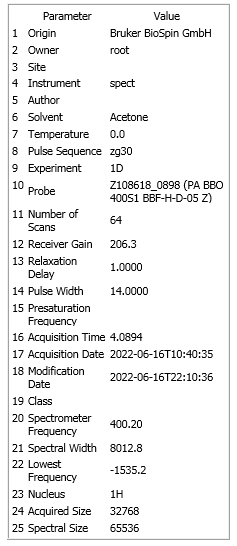


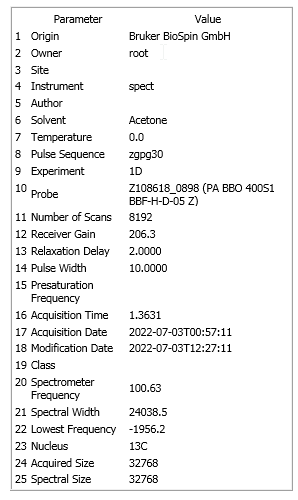


**
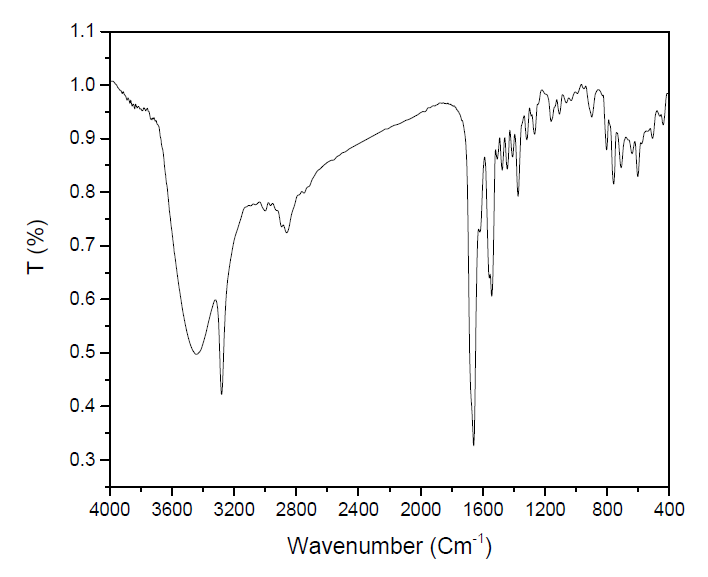
**


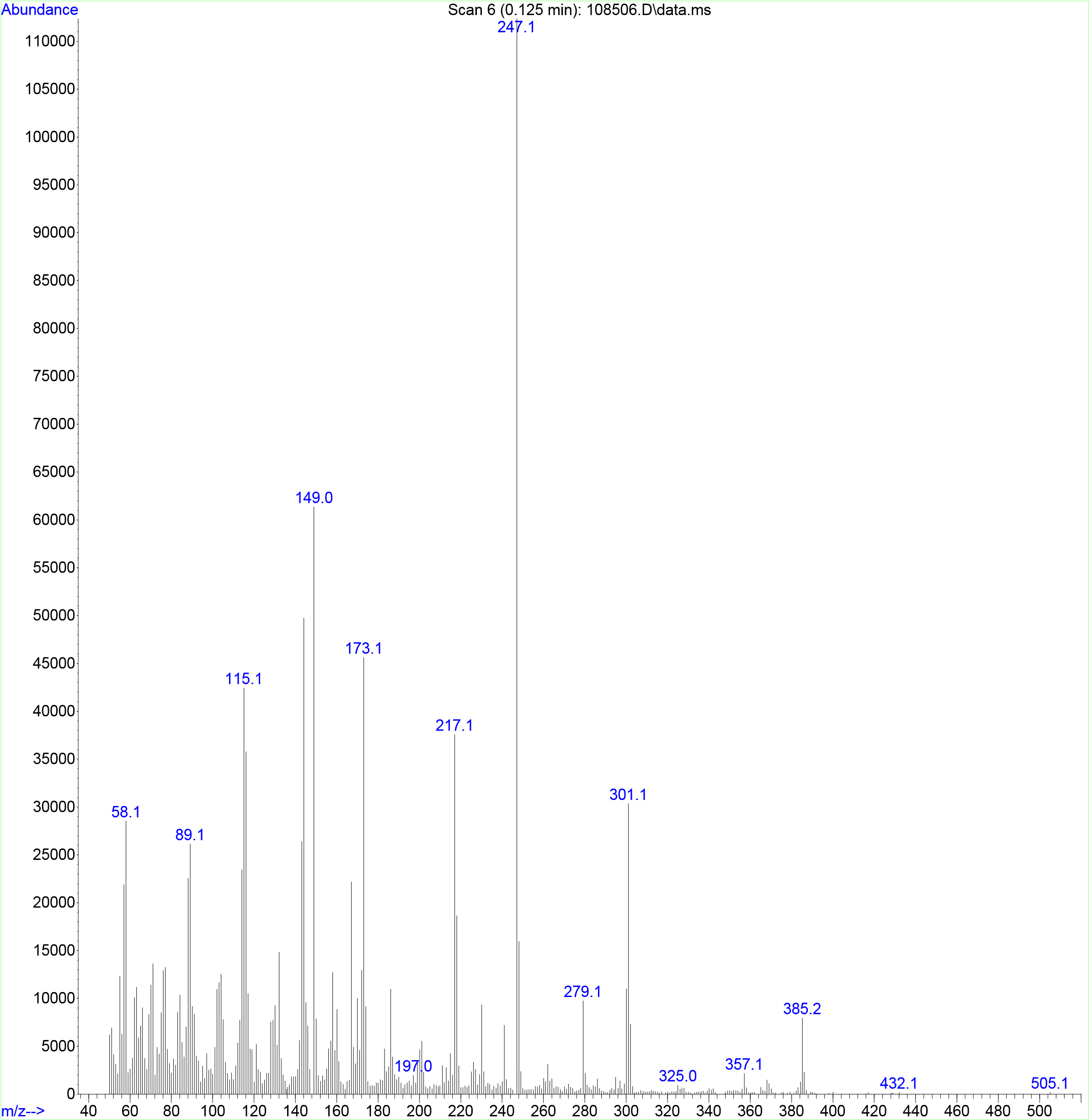


**
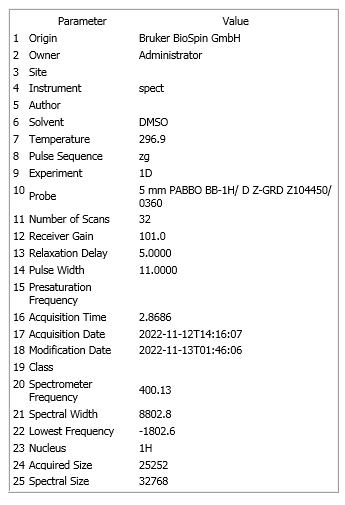
**

**
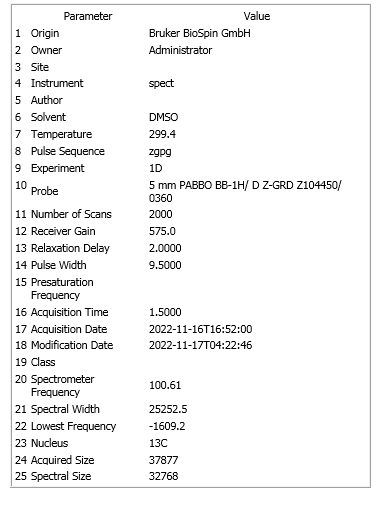
**
